# Biomarker Discovery for Meta-Classification of Melanoma Metastatic Progression Using Transfer Learning

**DOI:** 10.3390/genes13122303

**Published:** 2022-12-07

**Authors:** Jose Marie Antonio Miñoza, Jonathan Adam Rico, Pia Regina Fatima Zamora, Manny Bacolod, Reinhard Laubenbacher, Gerard G. Dumancas, Romulo de Castro

**Affiliations:** 1 System Modeling and Simulation Laboratory, Department of Computer Science, University of the Philippines Diliman, Quezon City 1101, Philippines; 2 Center for Informatics, University of San Agustin, Iloilo City 5000, Philippines; 3 Department of Microbiology and Immunology, Weill Cornell Medicine, New York, NY 10065, USA; 4 Department of Medicine, University of Florida, Gainesville, FL 32610, USA; 5 Loyola Science Center, Department of Chemistry, The University of Scranton, Scranton, PA 18510, USA; 6 3R Biosystems, Long Beach, CA 90840, USA

**Keywords:** melanoma, biomarker, transfer learning, ensemble model, bias, machine learning

## Abstract

Melanoma is considered to be the most serious and aggressive type of skin cancer, and metastasis appears to be the most important factor in its prognosis. Herein, we developed a transfer learning-based biomarker discovery model that could aid in the diagnosis and prognosis of this disease. After applying it to the ensemble machine learning model, results revealed that the genes found were consistent with those found using other methodologies previously applied to the same TCGA (The Cancer Genome Atlas) data set. Further novel biomarkers were also found. Our ensemble model achieved an AUC of 0.9861, an accuracy of 91.05, and an F1 score of 90.60 using an independent validation data set. This study was able to identify potential genes for diagnostic classification (C7 and GRIK5) and diagnostic and prognostic biomarkers (S100A7, S100A7, KRT14, KRT17, KRT6B, KRTDAP, SERPINB4, TSHR, PVRL4, WFDC5, IL20RB) in melanoma. The results show the utility of a transfer learning approach for biomarker discovery in melanoma.

## 1. Introduction

Melanoma is a cancer arising from pigment-containing cells called melanocytes. It is considered to be the most serious and aggressive type of skin cancer [1,2]. Its etiology is influenced by both genetics and environmental factors [3,4,5]. Prior to its diagnosis, melanoma has often spread to a distant location [6]. Therefore, the majority of deaths related to this disease is caused by its metastases.

Metastases appear to be the most significant factor influencing melanoma patients’ prognosis. Therefore, the advancement of new therapeutic strategies to extend patients’ overall survival will benefit from research into the mechanisms of melanoma metastasis. Since the advent of new therapies and interventions, such as immune checkpoint inhibitors and targeted therapies for metastatic melanoma, mortality rates for melanoma have decreased by 6.4% per year in the United States from 2013 to 2017 [7,8]. To support these new treatments, novel molecular biomarkers that can be used for diagnosis, prognosis, and treatment selection are needed. These biomarkers may further reveal molecular mechanisms of melanoma metastasis that could aid in informing and improving patients’ overall survival.

Gene expression profiling has been a powerful tool for identifying biomarker molecules involved in melanoma metastasis [9,10]. To leverage this, machine learning techniques have been considered in cancer prognostic development as genomic data have become more accessible. Nevertheless, cancer prognosis remains extremely challenging due to the high dimensionality of the data and the small number of patient samples. Several machine learning techniques have already been used as a disease classifier in melanoma but are primarily focused on images [11,12,13,14,15,16] and single predictive modeling approaches [17,18,19]; genomic signatures which may be more informative and accurate have not been considered. Compared to other related investigations [1,2,20,21], this study proposes a transfer learning approach as a biomarker discovery technique, and ensembles various classifiers that operate on different identified genomic signature subsets by soft voting. In addition, the level of expression of the weighted genomic biomarkers was investigated, in terms of survival of the patients, for a better understanding of melanoma metastasis and for the identification of potential therapeutic targets. Finally, preliminary data assessments allowed us to make predictions regarding bias and model performance, for a better identification of the subsets of patients that the ensemble model could be applied to.

## 2. Materials and Methods

### 2.1. Transfer Learning for Biomarker Discovery

Machine learning algorithms that were developed to store the information acquired and applied to a different but related problem are referred to as transfer learning [22]. A large number of data and computing resources may be required to train a model, but transfer learning can possibly address this issue. As a result, using transfer learning for data sets with high dimensionality and potentially complex interactions could be beneficial.

**Figure 1:**
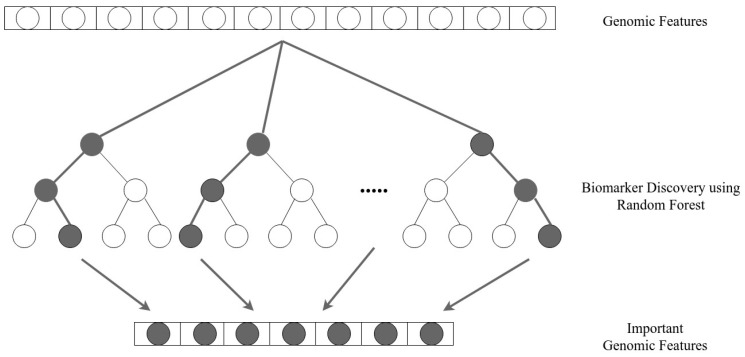
Biomarker discovery using random forest.

Biomarker discovery seeks to identify a subset of measured variables (i.e., genomic or clinical characteristics) that can be used to reliably predict a disease phenotype [23]. One of the popular approaches in transfer learning is feature extraction, which in this case, involves extracting genomic features possibly responsible for melanoma progression. Rule-based transfer learning for biomarker discovery was shown to have an improvement in its classification performance; however, it also has noticeable poor performance on structure learning [23]. On the other hand, random forest appears to be effective at finding interesting features in high-dimensional phenotype data with small key effects and low heritability [24]. This may be due to the way it accounts for potential gene–gene interactions when calculating significance scores for specific attributes (Figure 1).

### 2.2. Protein–Protein Interaction Network

The complex interactions of all molecules describe biological processes best and determine various cellular functions and responses. Mapping is a crucial step in trying to unravel their unique molecular relationships in specific biological contexts and eventually targeting therapy for treatment of diseases, such as cancer [25,26,27]. In mapping, protein–protein interaction (PPI) networks are typically represented as graphs, with nodes representing proteins, and edges connecting pairs of interacting proteins that are undirected and presumably weighted [28]. In contrast to traditional feature selection techniques, biomarker discovery using transfer learning could inform us of significant genomic features through computational methods. However, it is also important to identify the nuances of biomarkers’ roles and their interactions with other genes. Therefore, identified genomic signatures as potential biomarkers were mapped into the whole network, and the PPI network was then acquired. The PPI information used in this study was downloaded from STRINGDB (https://string-db.org/api/tsv/network, accessed on 15 March 2021), a database containing protein interactions that include physical and functional associations [29]. To identify which genes hold the most information, betweenness centrality was used, then the genes were ranked according to the following equation:

(1) $$g\left(v\right)=\underset{s\neq v\neq t}{\sum }\frac{\sigma_{st}\left(v\right)}{\sigma_{st}}$$

where *v* is the node gene retrieved from STRINGDB, *σ_st_* is the total number of shortest paths from node gene *s* to node gene *t*, and *σ_st_*(*v*) is the number of those paths that pass through *v*.

### 2.3. Clinical and Genomic Data

RNA-seq and clinical data for skin cutaneous melanoma (SKCM) were retrieved from The Cancer Genome Atlas (TCGA) using the TCGAbiolinks R package [30,31]. The data set contains 365 metastatic and 103 primary tumor samples. Then, the normalized read counts (per million reads mapped) of RNA-seq underwent log_2_ transformation (i.e., all values less than 1 were assigned to 1 before transformation). Thereafter, we carried out normalization of the data since the level of expression of genes varied in different scales. To reduce low variance features, 0.95 was set as variance threshold, which led to the decrease in genomic features from 19,947 to 19,815 counts for training the machine learning model. The data were randomly stratified into training (70%) and validation sets (30%), 286 and 123 patients, respectively.

In this study, the underlying bias within the data set was assessed to ensure that the end users of the models are aware of the potential shortcomings when applied in the clinical setting (once validated).

#### 2.3.1. Machine Learning Models

Machine learning techniques have been used in a wide variety of medical applications. However, they are commonly used on imaging data, such as ultrasound, X-rays, and slide specimens [32,33,34]. Similarly, in melanoma [35,36], computer vision is naturally used since the disease is first suspected visually through skin lesions. However, according to one study [6], by the time melanoma is discovered, it has already metastasized. This study attempts to develop a meta-classification model that can determine late stage (metastasis) from early stage (primary tumor) melanoma using genomic data.

The biomarkers from both random forest (in Section 2.1 and Section 2.2), through feature importance scores, and PPI network, through betweenness centrality scores, were rank selected and applied to (i) logistic regression, (ii) support vector machines, (iii) Gaussian Naïve Bayes, and (iv) random forest. In classification models, such as those used for identifying the melanoma stage, the Area Under the Receiver Operating Characteristic Curve (AUC) provides the probability that a randomly selected melanoma patient with metastatic stage will have a higher predicted probability of being metastatic than a randomly selected melanoma patient with a primary tumor stage.

DeLong’s method [37] was used to compare the performance of two models and accounted for the uncertainty caused by the finite training set randomness and the evaluation on a common validation set. To calculate the *z*-score when comparing models *A* and *B* in terms of AUC, the following equation was used:

(2) $$z≜\frac{{\hat{\theta }}^{\left(A\right)}-{\hat{\theta }}^{\left(B\right)}}{\sqrt{V\left[{\hat{\theta }}^{\left(A\right)}-{\hat{\theta }}^{\left(A\right)}\right]}}=\frac{{\hat{\theta }}^{\left(A\right)}-{\hat{\theta }}^{\left(B\right)}}{\sqrt{V\left[{\hat{\theta }}^{\left(A\right)}]+V[{\hat{\theta }}^{\left(B\right)}]-2C[{\hat{\theta }}^{\left(A\right)},{\hat{\theta }}^{\left(B\right)}\right]}}$$

where ${\hat{\theta }}^{\left(A\right)},{\hat{\theta }}^{\left(B\right)}$ are AUC scores of models *A* and *B*, respectively, *V* is the variance, and *C* is the covariance function. Under the null hypothesis [38], *z* can be well approximated by the standard normal distribution. Therefore, if the value of *z* deviates significantly from zero (e.g., *z* > 1.96), then it is rational to consider that ${\hat{\theta }}^{\left(A\right)}>{\hat{\theta }}^{\left(B\right)}$ at the significance level *p* < 0.05; namely, if *z* deviates significantly from zero, we can infer that model *A* has a statistically different AUC from model *B* at *p* < 0.05.

Rather than committing completely to a single best classifier, two or more models that appear to complement each other (e.g., models that perform exceptionally well in different regions of the Receiver Operating Characteristic (ROC) space) could be combined. Therefore, the models will be selected based on significant AUC scores and are ensembled via soft voting.

In soft voting [39], the predicted class labels based on the predicted probabilities *p* for each classifier are given by the following equation:

(3) $$\hat{y}={\mathrm{argmax}}_{i}\;\overset{m}{\underset{j=1}{\sum }}w_{i}p_{ij}$$

where *i* ∈ {0, 1} are class labels and $w_{i}$ is the weight that can be assigned to the *j*-th classifier. In this study, weights were uniform across the classifier models.

#### 2.3.2. Survival Analysis

In addition to disease diagnosis, we aimed to determine the disease prognosis, which deals with the probability of patient survival and time period. Since there were no machine learning techniques used in this methodology, the entire data set was used without data splitting.

A commonly used tool [40] for modeling and visualizing patient survival is the Kaplan-Meier analysis [41]. Within the context of melanoma, the Kaplan-Meier curve describes the survival rate or the number of melanoma patients surviving at each time point from diagnosis as given by the following survival function:

(4) $$\hat{S}\left(t\right)=\underset{t_{i}<t}{\prod }\left(\frac{n_{i}-d_{i}}{n_{i}}\right)$$

where *t* is the elapsed time after diagnosis, *d* is the number of death events at time *t*, and *n* is the number of melanoma patients at risk at time *t*.

Davidson-Pilon Lifelines KaplanMeierFitter (KMF) [42] Python module was used to estimate the survival function in Equation (4) and the survival curves were plotted. The KMF module required two inputs, event E and duration T, for which the patient was observed for event E. We used the ‘vital_status’ field from TCGA as event E, in order that a value of one (1) indicates death was observed while a value of zero (0) indicates right-censoring (loss to follow-up). For input T, we created another field, ‘days_to_event,’ which is a combination of the ‘days_to_death’ and ‘days_to_last_follow_up’ fields of the TCGA data set, in order that the empty values in the ‘days_to_death’ field are filled with ‘days_to_last_follow_up.’

Two Kaplan-Meier curves can be plotted on the same graph to determine whether a certain variable (e.g., age, gender) produces statistically different survival rates. In this study, we aimed to determine whether certain genes (i.e., variable of interest) affect the prognosis of melanoma patients; namely, whether a patient with high expression of a certain gene would yield poor survivability or whether a patient with low expression of a certain gene would yield better survivability. After normalizing the data using the standard scaling per gene, we used the statistical mean as the threshold for high and low gene expressions. Log-rank test with *α* = 0.99 indicates that if the *p*-value is less than 0.005 for a certain gene, then the two Kaplan-Meier curves are statistically different, and, therefore, the gene is a potential driver of prognosis.

## 3. Results

The main objective of this study was to identify expression signatures that can separate primary and metastatic SKCM based on RNA-seq expression data. After categorical features, such as race, gender, ethnicity, and vital status were converted by One Hot Encoding, the category Black and African American in the race data field was dropped since it has only one record and cannot be represented in both the training and validation data sets. Then, each of the models was fine-tuned via grid-search scorings using accuracy and F1 scores.

In selecting biomarkers, each of the trees in random forest was built over a random extraction of patient observations from the TCGA data set and a random extraction of the genomic features. Since not every tree observes all of the characteristics or all of the findings, the trees are de-correlated and, therefore, less vulnerable to overfitting. Each tree estimator has a series of true or false questions based on the level of expression of each of the genes and divides the observations based on their respective similarities and differences. Therefore, the ranking of importance of each gene was derived from how pure they are. The measure of impurity used in this study is the Gini impurity. For a deeper understanding, features selected at the top of the trees are usually more important than features selected at the end nodes of the trees, since top splits generally result in larger knowledge gains.

Random forest was first trained with 19,815 genes and was fine-tuned using a grid-search method to find the optimal hyperparameters. The random forest model used for biomarker discovery has the following hyperparameters: Maximum features of 60%, minimum samples of each leaf equal to 8, and the number of estimators equal to 30. Then, the feature importance of the model was analyzed. Table 1 shows the Top 30 genes of 139 that were found to be significant (i.e., weighted).

**Figure 2:**
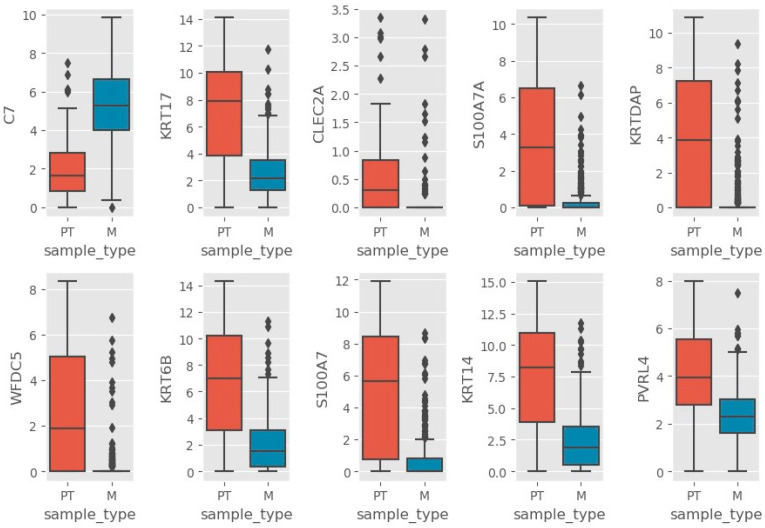
Box plots of Top 10 gene expressions. M refers to metastatic and PT refers to primary tumor.

The expression of the Top 10 genes obtained from random forest as potential biomarkers was further examined (Figure 2). C7 is upregulated in metastatic sample compared to primary tumor, while KRT17, CLEC2A, S1007A, KRTDAP, WFDC5, KRT6B, S100A7, KRT14, and PVRL4 are downregulated (or upregulated in primary tumor compared to metastatic sample). The rest of the genes showing significant (*p <* 0.05, Welch’s *t*-test, see Supplementary Materials File S10) upregulated expression in primary tumor or metastatic sample type are shown in Table 2.

As can be seen from the extracted genomic features, there are genes of related functionality, such as KRT17, KRTDAP, KRT6B, and KRT14. Random forest showed the genes that have a potential for classifying melanoma based on a specific gene expression. We hypothesized that it is important to identify genetic interactions, in order to derive the genes that hold the most information and leverage it to further improve the performance of the model. In developing a model, this can be viewed as optimizing the bias variance trade-off, wherein high bias can miss possible relevant genes (underfitting) and high variance may include multicollinear genomic features (overfitting) in the model.

Of the 139 genomic features identified by random forest, 22 genes were found to be highly connected with other genes on the list. Among these 22 information-heavy genes, C7, S100A7, SERPINB4, GRIK5, KRT14, PAX1, and KRT6B figured prominently in feature selection (Table 1), while genes, such as PC, RPN2, TSHR, GSR, RPS28, and GNG2 which were not as prominent have risen to the top (Table 3).

### 3.1. Model Performance

During the model tuning, F1 and accuracy scores were used as metrics to improve the performance since there is an imbalanced class in the data set. AUC score was used as the final metric since it is commonly used to depict the trade-off relationship between clinical sensitivity and specificity for each potential cut-off for a test or a set of tests in a graphical format. Moreover, AUC provides insight into the value of using the model in diagnosing melanoma patients. Furthermore, it determines how well the model correctly classifies a metastatic melanoma patient given the yield probability that the patient indeed has metastatic melanoma.

The model performance was compared for the top genes, progressively selected (Top 10, Top 20, Top 30, Top 40, Top 50 as in [1]), using logistic regression, support vector machines (linear, polynomial, radial basis, and sigmoid kernel), Gaussian Naïve Bayes, and random forest models. These models were trained using a 5-fold cross validation.

Overfitting occurs as performance in the training set improves but performance on the validation or test data set worsens; therefore, the determination of the best number of genes for a specific algorithm is conducted via the performance gap between the training and validation set. Unfortunately, support vector machines with polynomial kernel do not perform well with the 139 genes identified [see Supplementary Materials, Files S3 and S4]. Table 4 shows the six best models and their validation scores that achieved high AUC scores. Based on the results, only the Top 30 of 139 identified genes were found to be important in diagnosing melanoma.

Similarly, in the PPI mapped genes that were ranked using betweenness centrality, the genes were progressively selected (Top 10, Top 20) and the performance was compared. Table 5 shows that logistic regression, support vector machines, and Gaussian Naïve Bayes achieved high validation AUC scores [See Supplementary Materials, Files S5 and S6]. To further investigate the performance of the models in terms of their AUC scores, De Long’s test [37] was conducted. It was found that RF-LR Top 10, RF-RF Top 20, and RF-PPI-SVM-Sig Top 10 models were significantly better (*p <* 0.05) [See Supplementary Materials, File S7]. The rank of random forest selected features might still miss some relevant genes and the rank using betweenness centrality might also increase the variance estimates across the samples. This can be further supported by the analysis on bias-variance decomposition among these models, found in Table S2, showing that RF-LR Top 10, RF-RF Top 20, and RF-PPI-SVM-Sig Top 10 models’ expected loss were minimized as bias and variance were optimized. Finally, the three significant models were combined as an ensemble model through soft voting. The resulting ensemble model still has high and acceptable validation scores (F1 = 90.60, Accuracy = 91.05, AUC Score = 0.9861, see Supplementary Materials, Table S2), after ensuring that the bias and variance were minimized. The unique gene signatures that were used in the ensemble model are listed in Table 6.

### 3.2. Kaplan-Meier Survival Analysis

We performed the Kaplan-Meier survival analysis on the 139 significant genes (Table 2) selected by random forest. Log-rank test identified 26 of the 139 genes that displayed a significant difference (*p*-value < 0.05) in terms of survival between high and low gene expressor.

**Figure 3:**
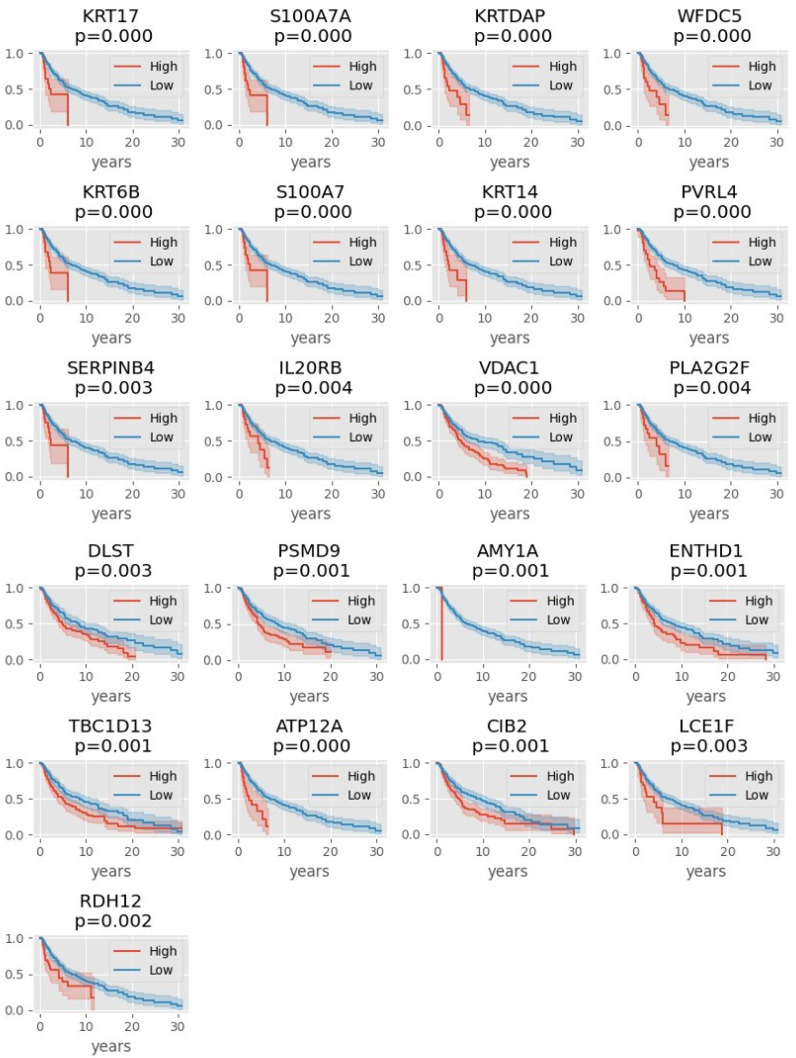
Kaplan-Meier plots showing that the red lines (high expressor) fall faster than the blue lines (low expressor) for each gene. The *y*-axis represents the probability of survival, thus, high expressors (red line) of these 21 genes have worse survival. In this analysis, the patient samples were divided in terms of the average of gene expression (high = above the mean, low = below the mean).

Our analysis shows that high expression of KRT17, S100A7A, KRTDAP, WFDC5, KRT6B, S100A7, KRT14, PVRL4, SERPINB4, IL20RB, VDAC1, PLA2G2F, DLST, PSMD9, AMY1A, ENTHD1, TBC1D13, ATP12A, CIB2, LCE1F, or RDH12 is associated with worse survival (Figure 3).

**Figure 4:**
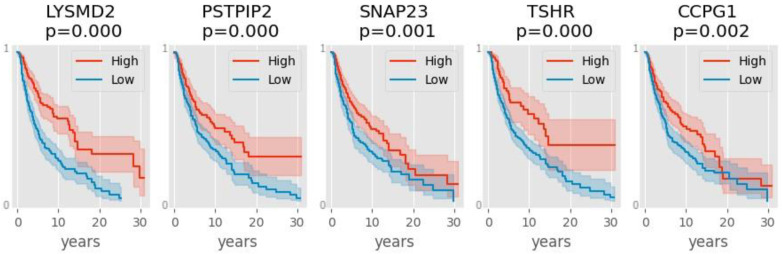
Kaplan-Meier plots showing that the blue lines (low expressor) fall faster than the red lines (high expressors) for each gene. The *y*-axis represents the probability of survival, thus, high expressors of these five genes have better survival. In this analysis, the patient samples were divided in terms of the average of gene expression (high = above the mean, low = below the mean).

On the other hand, high expression of LYSMD2, PSTPIP2, SNAP23, TSHR, or CCPG1 is associated with better survival (Figure 4). Herein, 11 of these 26 prognostic genes, *KRT17*, *S100A7A*, *KRTDAP*, *WFDC5*, *KRT6B*, *S100A7*, *KRT14*, *PVRL4*, *SERPINB4*, *IL20RB* and *TSHR*, are common with the 26 genes identified by the ensemble classifier (Table 6).

### 3.3. Implicit Bias

The evaluation of data is a critical step in the development of machine learning models, especially when they are used in clinical decision support for medical diagnosis. Initial exploratory analyses show that training the model with the TCGA data for melanoma has implicit bias on race, gender, age groups, and Body Mass Index (BMI). There were more metastatic samples analyzed compared to primary tumor; patients were concentrated in the 40–79 age group; the female to male ratio was 0.58 (Figure S2); in terms of race, white patients were dominantly present in the data set (Figure S3); and for BMI, underweight patients were not represented (Figure S4). We hypothesized that our machine learning model will only perform well on populations that are well represented demographically.

We calculated the performance accuracy on sample type, age groups, gender, BMI, and race using the ensemble model. Results shown in Table 7 confirmed our hypothesis for sample type and age group, but not for gender and race where the model still performs quite well despite the unevenness of the data. For the 0–19 age group, the perfect performance of the model is likely an overfit due to the very small number of samples.

## 4. Discussion

Feature selection in machine learning applied to gene expression data is a powerful method that can identify biomarkers to classify disease states (primary vs. metastatic melanoma as in this study). Once the list of potential biomarkers is narrowed down and ranked for their respective contributions (139 weighted genes ranked), additional machine learning methods, such as logistic regression, support vector machine, and Gaussian Naïve Bayes can further indicate which rank cut-off is important based on model performance (as in this case, Top 30), providing a more manageable set of molecular markers for further study.

Furthermore, PPI analysis of the original 139 genes in the random forest learning set, showed that lower ranked genes can figure prominently, indicating that interactions may be important. Of the Top 10 genes in betweenness centrality score, only C7 and S100A7 were also in the random forest Top 10 (see Table 1), yet the performance of models incorporating the Top 10 PPI-selected genes was still very high (0.935–0.9552, Table 5) even though PC, RPN2, TSHR, GSR, RPS28, and GNG2 genes were not in the RF Top 30.

Comparison of the performance of several models distinguished the best three models, which when ensembled, in order not to miss other relevant genes, performed very well (AUC = 0.9861, Supplementary Materials, Table S2). There are 26 genes in the ensemble meta-classifier, including genes involved in skin cell differentiation (CLEC2A, KRT6B, KRT14, KRT17, KRTDAP), immunity (S100A7, S100A7A, IL20RB, C7, PRG2, SERPINB4, WFDC5, FKBP1B), cell adhesion (PVRL4), energy/metabolism (PC, TSHR), cancer metastasis (AFAP1-AS1) and suppression (DMBT1), cellular redox (GSR), cell signaling (GNG2), cell division (ZSWIM7), protein synthesis and modification (RPS28, RPN2), and transcriptional regulation (ZNF653, PAX1). Moreover, the majority of these genes have been linked to other cancers; therefore, the methods we employed found genes involved in metastatic progression which could be common among cancers. Interestingly, the Glutamate Ionotropic Receptor Kainate Type Subunit 5 (GRIK5) identified here for the first time as a classifier for primary vs. metastatic melanoma, is mainly known for its role in neural development and neuropsychiatric disorders [43,44,45].

Examining the profiles of the 139 genes in patient tissue, we found that some of these genes are highly expressed in metastatic tissue compared to primary tumor, such as C7, DOCK11, SCN4A, etc. However, more genes in this set were expressed highly in primary tumors (Table 2). Genes, such as members of the keratin family (KRT17, KRTDAP, KRT6B, KRT14, KRTAP13-2) are expressed more in primary tumor, possibly indicating the differentiated status of less advanced cancers, or this could be a disruption in their normal expression by melanoma processes. (Unfortunately, we could not compare expression with normal skin tissue since none of these samples were included in the data set).

When expression of these genes was correlated with patient survival, we found genes whose high expression correlate with worse (Figure 3) or better (Figure 4) survival. Some of the genes that were highly expressed in primary tumors (such as the keratin genes) turned out to be predictive, but, oddly, of poor outcome. We can only surmise that possibly the early stage of melanoma increases the expression of these genes, but this disruption may be detrimental to the patient eventually. Only five genes were found to be predictive of good outcome when highly expressed in melanoma: LYSMD2, PSPIP2, SNAP23, TSHR, and CCPG1. Of these, only TSHR was identified by the ensemble classifier. Thyroid Stimulating Hormone Receptor (TSHR) controls thyroid cell metabolism, and defective TSHR causes hyperthyroidism. The expression of this hormone receptor has been observed in melanoma [46]; its downregulation has been associated with thyroid cancer metastasis and is prognostic for poor survival [47], in agreement with our findings.

Moreover, TSHR has been identified for therapeutic intervention or as a theranostic indicator for thyroid, ovarian, and hepatic malignancies [48], demonstrating the utility of our methods in the identification of potential therapeutic targets for oncology. Very little is known about LYSMD2 and PSPIP2, but Synaptosome-Associated Protein 23 (SNAP23) is a vesicular transport protein that is highly expressed in lymph nodes and the spleen (https://www.ncbi.nlm.nih.gov/gene/8773) pointing to a possible involvement in immunity. Cell Cycle Progression 1 (CCPG1) is involved in endoplasmic reticulum homeostasis [49] and may be a tumor suppressor gene [2].

Assessing the data for potential bias is a recommended exercise and should be continuously conducted during artificial intelligence implementation, in order to correct for under or over representation of specific populations in machine learning, and to help interpret model performance. The ultimate goal is to be able to roll out a fairer algorithm, which, if used in health, would not result in further inequities as is machine learning’s wont. The analysis predetermines the segments of the patient population that our models would likely work in. According to our assessments, the TCGA SKCM data set is biased on sample type (metastatic > primary tumor), age (40–79-year-olds are best represented), gender (male > female), and race (mostly white, few Asians, and no other race categories). We expected our final model to perform best in the most represented groups, which it did in terms of sample type and age. Surprisingly, the model still performed robustly with respect to gender and race. Moreover, it must provide tolerance down to a gender ratio of 0.58 (female to male). However, it is very interesting that even with an extreme race ratio of 0.04 (Asian to white), the model still works albeit with somewhat lowered performance. We dropped the single black patient for this analysis; thus, we cannot generalize this model to the black population. The lack of data may be a reflection of the relatively low incidence of melanoma in the black population. For BMI, the segments are fairly represented, with no underweight patients. Consequently, the model performed well in all BMI segments, but, again, it may not be extendable to underweight patients.

### 4.1. Implicit Bias

Our models were able to identify notable genes, specifically ones also flagged by survival analysis. Some of these genes have been identified in previous studies involving machine learning on the same data set [20,21]. Moreover, these genes are good candidates for validation ahead of their potential applications in the clinical setting.

### 4.2. Potential Diagnostic Classifiers

**C7.** C7 is a member of the soluble Membrane Attack Complex (sMAC), along with C5b, C6, C8, and C9, which is generated upon activation of the complement system [50]. In a study performed by Bhalla et al. (2019) that used several feature selection methods on genomic data, C7 figured prominently in melanoma carcinogenesis and was also found to be upregulated in metastatic tumors [21]. Opposing observations were seen among ovarian and Non-Small Cell Lung Cancer (NSCLC) tissues as C7 was found to be further downregulated as the tumor stage increased. More importantly, low C7 levels were also identified to be a significant prognostic factor for NSCLC patients [51]. The inclusion of C7 as a diagnostic classifier to distinguish between primary and metastatic melanoma is promising and warrants further investigation.

**GRIK5.** GRIK5 encodes for the kainate-preferring glutamate receptor subunit KA2, which is ubiquitously expressed in the mammalian brain [52]. In the SKCM data set, the expression of GRIK5 does not seem to be significantly different between primary and metastatic melanoma. However, in preliminary studies on zebrafish, decreased expression of GRIK5 was found to lead to vascular pathologies in the eye and brain. Moreover, they have been shown to be associated with patterning and vasculature integrity [53]. Given the earlier observations, the potential role of GRIK5 in angiogenic processes necessary for metastasis warrants further investigation.

#### Potential Diagnostic and Prognostic Biomarkers

**S100A7/S100A7A.** In this study, S100A7 was shown to be upregulated in primary melanoma. Analysis of publicly available gene expression profiles showed that S100A7 was highly expressed in primary cutaneous melanoma, but was significantly decreased in normal skin tissue and metastatic melanoma. A follow-up analysis of PPI identified S100A7 as a hub gene in primary cutaneous melanoma [54].

At the transcriptomic level, a study performed by Riker et al. (2008) showed that S100A7 expression was highly expressed in primary cutaneous melanoma vs. normal skin tissue but was seen to significantly decrease in metastatic melanoma [10]. A similar study showed that several S100 family genes, including S100A7, were highly expressed in primary melanoma, but were seen to significantly decrease in metastatic melanoma [55]. More importantly, higher levels of S100A7 were detected in the urine of cutaneous melanoma patients compared to a control group. In addition, this trend was not seen in a heterogeneous group of patients with other cancer types [56]. The significant levels of S100A7 expression in primary cutaneous melanoma and the ease in detection in urine samples make it a promising diagnostic classifier.

**KRT14, KRT17, KRT6B, KRTDAP.** These genes are involved in keratinization. Increased expression of KRT6B, KRT14, and KRT17 was associated with poor survival in melanoma [57]. On the other hand, KRTDAP was found to have higher expression in primary tumor compared to metastatic tumor [10]. The role of KRTDAP is mainly in keratinocyte differentiation; therefore, this may indicate that metastatic melanoma tissue is less differentiated compared to primary lesions.

**SERPINB4.** The squamous cell carcinoma antigen 2, encoded by the genes SERPINB3 and SERPINB4, has been shown to be involved in inflammatory conditions of the skin and respiratory diseases, such as chronic obstructive pulmonary disease and tuberculosis [58]. It has been used as a diagnostic marker for advanced squamous cell carcinoma in the head and neck [59]. Moreover, it has been found to induce Epithelial-Mesenchymal Transition (EMT) in mammalian epithelial cells, insinuating its role in tumor metastasis [60].

**TSHR.** TSHR has been documented to be expressed in all melanocytic lesions, with higher levels found in malignant and pre-malignant lesions. Its ligand, the thyroid stimulating hormone, was found to induce melanoma proliferation. Circulating levels of TSH increase in thyroid failure conditions providing an environment where melanoma can proliferate [61]. In the clinical setting, it was found that patients with cutaneous malignant melanoma were at a higher risk for other cancers, especially thyroid carcinoma [62].

**PVRL4.** PVRL4, also known as NECTIN4, was identified as a potent inducer of anchorage-independent growth in epithelial cell culture [63]. In cancer, an increased expression of PVRL4 was found to be associated with high-grade serous ovarian carcinoma but did not seem to be involved in survival [64].

**WFDC5.** WFDC5 is highly expressed in human epidermis [65], which is known to secrete protease inhibitors involved in inflammatory processes [66]. It was found to be upregulated in head and neck squamous cell carcinoma expression data from the GEO database [54]. Using microarray data, WFDC5 figured in the Top 40 of a candidate 200-gene signature, which is able to distinguish between melanoma and normal epithelial cells/benign nevus [67].

**IL20RB.** IL20RA and IL20RB are subunits of the Interleukin 20 Receptor Type I (IL20RI) found in the epidermis [68,69]. IL20RB was found to be associated with inflammatory processes in psoriasis [70] and vitiligo [71]. IL20RB expression levels have already been documented in several cancers. Cui et al. (2019) showed that it is highly expressed in Papillary Renal Cell Carcinoma (PRCC) tissue and was linked to poor prognosis among patients. In the same study, its repression limited the proliferation and migration of PRCC cells; therefore, highlighting its potential role in the EMT mechanisms leading to metastasis [72]. This finding can be corroborated by the function of one of the IL20R1, IL20RA + IL20RB)/IL20R2 heterodimer ligands, IL20, which is a pro-inflammatory cytokine found to enhance wound healing, migration, and invasion in bladder cancer cell lines [73]. This evidence points to the potential role of IL20RB in inflammatory processes in melanoma, which warrants further investigation.

## Figures and Tables

**Table 1:** The 30 genes exhibiting the highest scores through random forest for biomarker discovery analysis.

Rank	Gene Code	Gene Name	Score
1	C7	Complement C7	0.1591
2	KRT17	Keratin 17	0.1029
3	CLEC2A	Keratinocyte-Associated C-Type Lectin	0.0912
4	S100A7A	S100 Calcium-Binding Protein A7A	0.0646
5	KRTDAP	Keratinocyte Differentiation-Associated Protein	0.0604
6	WFDC5	WAP Four-Disulfide Core Domain 5	0.0418
7	KRT6B	Keratin 6B	0.0389
8	S100A7	S100 Calcium-Binding Protein A7 (Psoriasin 1)	0.0242
9	KRT14	Keratin 14	0.0196
10	PVRL4	Nectin Cell Adhesion Molecule 4	0.0176
11	SERPINB4	Squamous Cell Carcinoma Antigen 2	0.0172
12	IL20RB	Interleukin 20 Receptor Subunit β	0.0114
13	AFAP1-AS1	AFAP1 Antisense RNA 1	0.0109
14	FKBP1B	FKBP Prolyl Isomerase 1B	0.0103
15	ZSWIM7	Zinc Finger SWIM-Type Containing 7	0.0094
16	PRG2	Proteoglycan 2, Pro Eosinophil Major Basic Protein	0.0091
17	PAX1	Paired Box Protein Pax-1	0.0087
18	DMBT1	Deleted In Malignant Brain Tumors 1	0.0086
19	ZNF653	Zinc Finger Protein 65	0.0085
20	GRIK5	Glutamate Ionotropic Receptor Kainate-Type Subunit 5	0.0081
21	MMP3	Matrix Metalloproteinase 3	0.0080
22	ZNF593	Zinc Finger Protein 593	0.0075
23	VDAC1	Outer Mitochondrial Membrane Protein Porin 1	0.0073
24	ADAMTSL3	ADAMTS-Like 3	0.0072
25	RGS4	Regulator Of G Protein Signaling 4	0.0071
26	MRPL44	Mitochondrial Ribosomal Protein L44	0.0070
27	LYSMD2	LysM Domain Containing 2	0.0068
28	TDRKH	Tudor And KH Domain Containing	0.0059
29	CSPG4	Melanoma-Associated Chondroitin Sulfate Proteoglycan	0.0057
30	PLA2G2F	Phospholipase A2 Group IIF	0.0056

**Table 2:** Upregulated genes according to sample type from among the 139 feature selected genes.

Primary Tumor	Metastatic
KRT17, CLEC2A, S100A7A, KRTDAP,	
WFDC5, KRT6B, S100A7, KRT14, PVRL4, SERPINB4, IL20RB, PAX1, MMP3, PLA2G2F, FCER1A, PSMD9, PRKRIP1, HMG20B, RAX, SSNA1, MRRF, PITHD1, COQ4, XKRX, FAM109B, C1orf159, MIEN1, RNF135, AKR1B15, SPSB3, SWI5, ATP12A, LCE1F, ALAD, FAAH, RDH12, RPS28, VDAC1, G6PC3, FAM98C, ZNF593, MRPL44, TBC1D13, ZSWIM7, PRG2, CICP27, CIB2, FKBP1B, ZNF653	C7, DOCK11, SCN4A, CLIC5, PDK4, SNAP23, PABPC4L, SMARCAL1, SAMD8, CCPG1, MRPL23, SLC9A8, TSPAN14, RARRES2, SLC40A1, GSR, IGF1R, DDX3X, PSTPIP2, CASK, SMTNL2, ADAMTSL3, ARHGAP22, RGS4, GTF2H2C, TAF5L, LYSMD2, TDRKH

**Table 3:** Betweenness centrality rank of genes from protein–protein interaction network.

Rank	Gene Code	Gene Name	Score
1	PC	Pyruvate Carboxylase, Mitochondrial	0.0886
2	RPN2	Ribophorin II	0.0636
3	TSHR	Thyroid Stimulating Hormone Receptor	0.0490
4	GSR	Glutathione Reductase, Mitochondrial	0.0396
5	RPS28	Ribosomal Protein S28	0.0370
6	GRIK5	Glutamate Ionotropic Receptor Kainate-Type Subunit 5	0.0185
7	GNG2	G Protein Subunit γ 2	0.0131
8	C7	Complement C7	0.0130
9	S100A7	S100 Calcium-Binding Protein A7 (Psoriasin 1)	0.0104
10	SERPINB4	Squamous Cell Carcinoma Antigen 2	0.0078
11	IGF1R	Insulin-Like Growth Factor 1 Receptor	0.0062
12	KRT14	Keratin 14	0.0061
13	NKX6-1	NK6 Homeobox 1	0.0052
14	MRRF	Ribosome-Recycling Factor, Mitochondrial	0.0051
15	RPE65	Retinoid Isomerohydrolase RPE65	0.0045
16	LMX1B	LIM Homeobox Transcription Factor 1 β	0.0032
17	PAX1	Paired Box Protein Pax-1	0.0032
18	PTF1A	Pancreas-Associated Transcription Factor 1a	0.0032
19	PTS	6-Pyruvoyltetrahydropterin Synthase	0.0026
20	KRT6B	Keratin 6B	0.0016
21	CASK	Calcium/Calmodulin Dependent Serine Protein Kinase	0.0013
22	FBXW10	F-Box and WD Repeat Domain Containing 10	0.0006

**Table 4:** Model performance (validation data set) using biomarkers discovered through random forest.

Model	Name	Genes	F1	Accuracy	AUC
RF-RF	Random Forest	Top 20	92.85	93.01	0.9789
RF-SVM-R	SVM (Radial Basis Kernel)	Top 10	86.60	87.76	0.9249
RF-LR	Logistic Regression	Top 10	87.59	88.81	0.9234
RF-NB	Naïve Bayes	Top 20	80.04	82.52	0.8252
RF-SVM-L	SVM (Linear Kernel)	Top 10	80.80	83.91	0.8205
RF-SVM-Sig	SVM (Sigmoid Kernel	Top 30	79.02	80.06	0.8054

**Table 5:** Model performance (validation data set) using biomarkers discovered through random forest and mapped by protein–protein interaction network.

Model	Name	Genes	F1	Accuracy	AUC
RF-PPI-SVM-L	SVM (Linear Kernel)	Top 20	83.15	85.36	0.9659
RF-PPI-NB	Naïve Bayes	Top 20	86.43	87.80	0.9054
RF-PPI-SVM-Sig	SVM (Sigmoid Kernel)	Top 10	73.17	79.67	0.9049
RF-PPI-LR	Logistic Regression	Top 10	83.15	85.37	0.8808

**Table 6:** The 26 genes in the ensemble meta-classifier with soft voting.

Gene Code	Gene Name	Location
S100A7	S100 Calcium-Binding Protein A7 (Psoriasin 1)	chr1
S100A7A	S100 Calcium-Binding Protein A7A	chr1
PVRL4	Nectin Cell Adhesion Molecule 4	chr1
FKBP1B	FKBP Prolyl Isomerase 1B	chr2
IL20RB	Interleukin 20 Receptor Subunit β	chr3
AFAP1-AS1	AFAP1 Antisense RNA 1	chr4
C7	Complement C7	chr5
GSR	Glutathione Reductase, Mitochondrial	chr8
DMBT1	Deleted In Malignant Brain Tumors 1	chr10
PRG2	Proteoglycan 2, Pro Eosinophil Major Basic Protein	chr11
PC	Pyruvate Carboxylase, Mitochondrial	chr11
CLEC2A	Keratinocyte-Associated C-Type Lectin	chr12
KRT6B	Keratin 6B	chr12
GNG2	G Protein Subunit γ 2	chr14
TSHR	Thyroid Stimulating Hormone Receptor	chr14
KRT14	Keratin 14	chr17
KRT17	Keratin 17	chr17
ZSWIM7	Zinc Finger SWIM-Type Containing 7	chr17
GRIK5	Glutamate Ionotropic Receptor Kainate-Type Subunit 5	chr19
KRTDAP	Keratinocyte Differentiation-Associated Protein	chr19
RPS28	Ribosomal Protein S28	chr19
ZNF653	Zinc Finger Protein 653	chr19
SERPINB4	Squamous Cell Carcinoma Antigen 2	chr18
PAX1	Paired Box Protein Pax-1	chr20
RPN2	Ribophorin II	chr20
WFDC5	WAP Four-Disulfide Core Domain 5	chr20

**Table 7:** Performance accuracy of the models to examine implicit bias in training data set.

Variable	N	Ensemble
Sample Type		
Primary Tumor	68	51.47%
Metastatic	218	99.54%
Age		
0–19	3	100.00%
20–39	29	89.66%
40–59	109	90.83%
60–79	124	87.09%
80+	21	71.19%
Gender		
Male	181	88.95%
Female	105	86.66%
BMI		
Normal	92	88.04%
Overweight	107	90.65%
Obese	86	84.88%
Race		
White	271	88.19%
Asian	10	80.00%

## Data Availability

The data used in this study were downloaded from TCGA (https://portal.gdc.cancer.gov/). The codes for this analysis can be found here: https://doi.org/10.5281/zenodo.4781962 (accessed on 15 March 2021).

## Supplementary Materials

The following supporting information can be downloaded at: https://www.mdpi.com/article/10.3390/genes13122303/s1. Figure S1: Distribution of sample type. Figure S2: Distribution of gender. Figure S3: Distribution of race. Figure S4: Distribution of BMI. Figure S5–S34: Biomarker discovery—random forest estimators. Figure S35–S40: Box plots of Top 139 gene expressions. Table S1: Bias variance decomposition of the models. Table S2: Model performance (validation data set) of ensemble model with soft voting. Files: File S1 feature selection (biomarker discovery) results from random forest, File S2 feature selection (biomarker discovery) results from random forest mapped to PPI using betweenness centrality, File S3 model evaluation scores for training data set [based on Top 139 genes of RF], File S4 model evaluation scores for validation data set [based on Top 139 genes of RF], File S5 model evaluation scores for training data set [based on Top 20 genes of RF-PPI], File S6 model evaluation scores for validation data set [based on Top 20 genes of RF-PPI], File S7 model comparison results using De Long’s test for AUC, File S8 implicit bias on training data set [SKCM], File S9 bias variance decomposition, File S10 Welch’s *t*-test results for 139 genes.
