# Supplementary material for: Biomarker Discovery for Meta-Classification of Melanoma Metastatic Progression Using Transfer Learning
Source: Genes (Basel). 2022 Dec 7;13(12):2303. doi: 10.3390/genes13122303 (PMC9777873; doi:10.3390/genes13122303)
Supplement: Supplementary file 1 [file genes-13-02303-s001.zip › Supplementary files/Supplementary_Materials.pdf]

Biomarker Discovery for Meta-Classification and Prognosis of Melanoma using Transfer Learning

1 Supplementary Materials

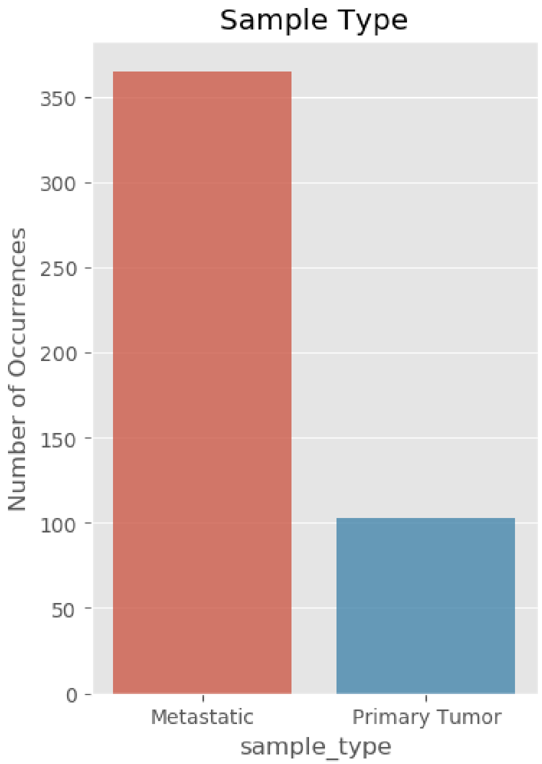

Figure S 1. Distribution of Sample Type

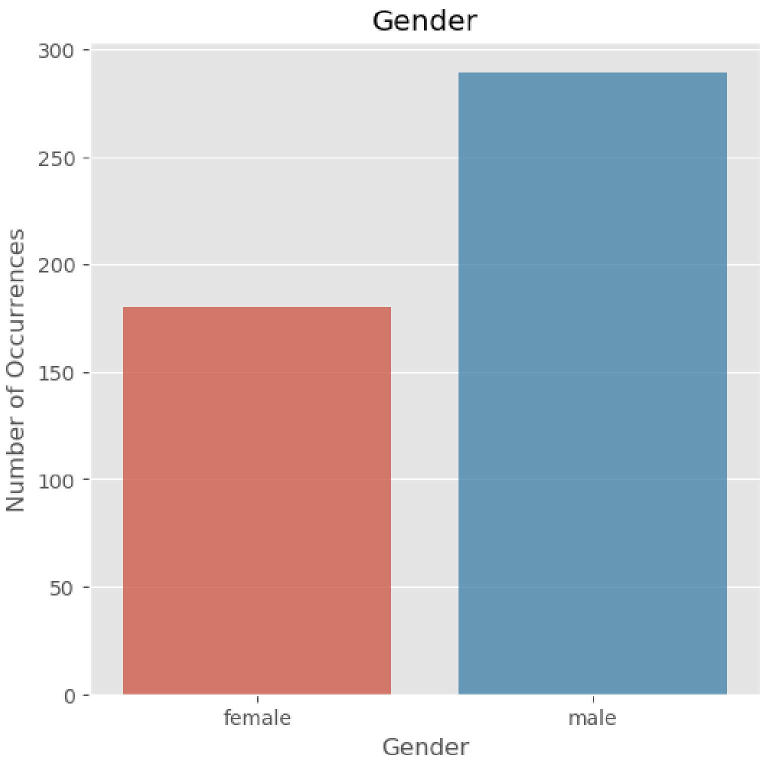

Figure S 2. Distribution of Gender

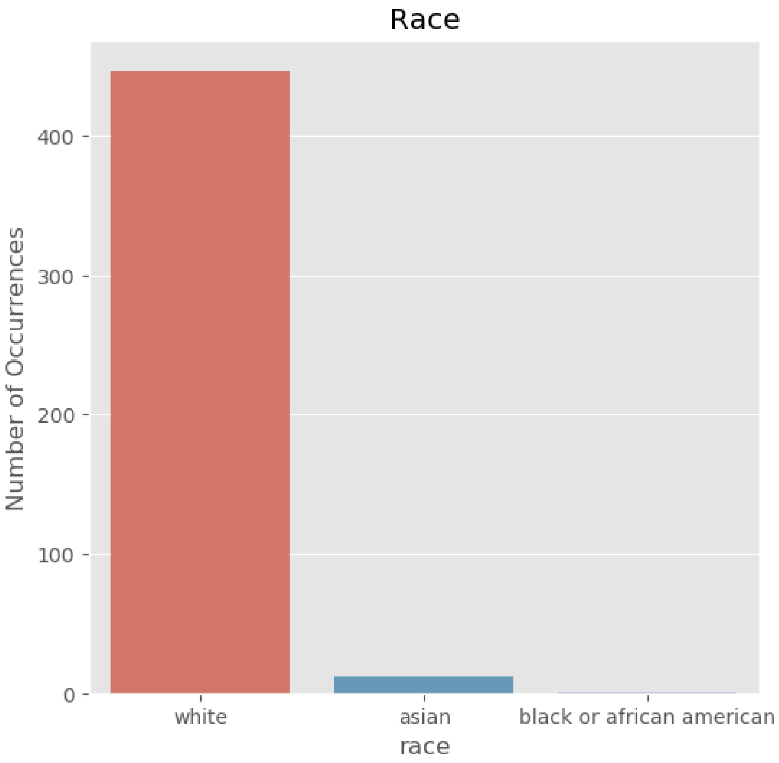

Figure S 3. Distribution of Race

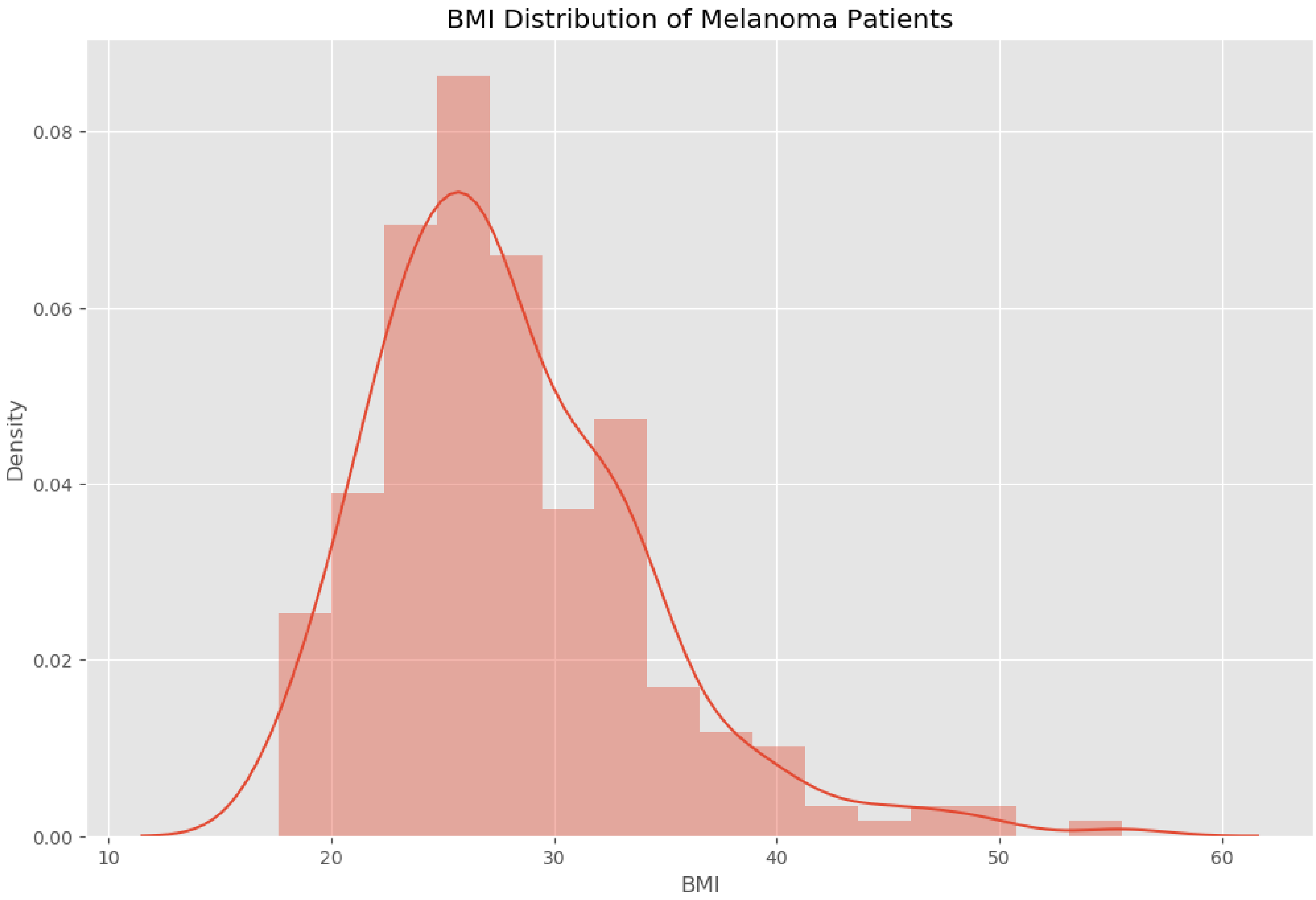

Figure S 4. Distribution of BMI

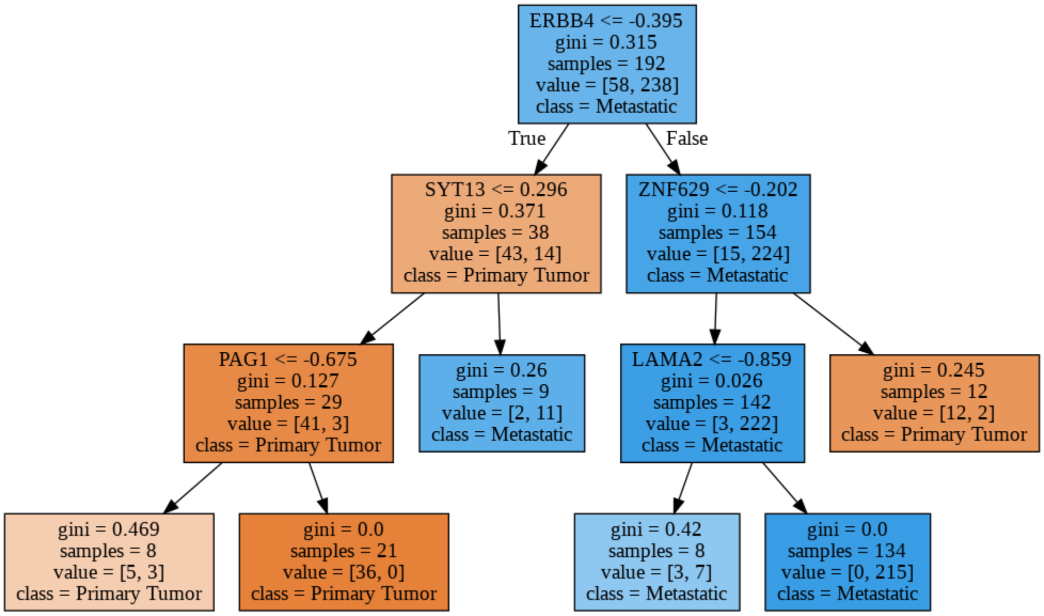

Figure S 5. Biomarker Discovery - Random Forest Estimator 1

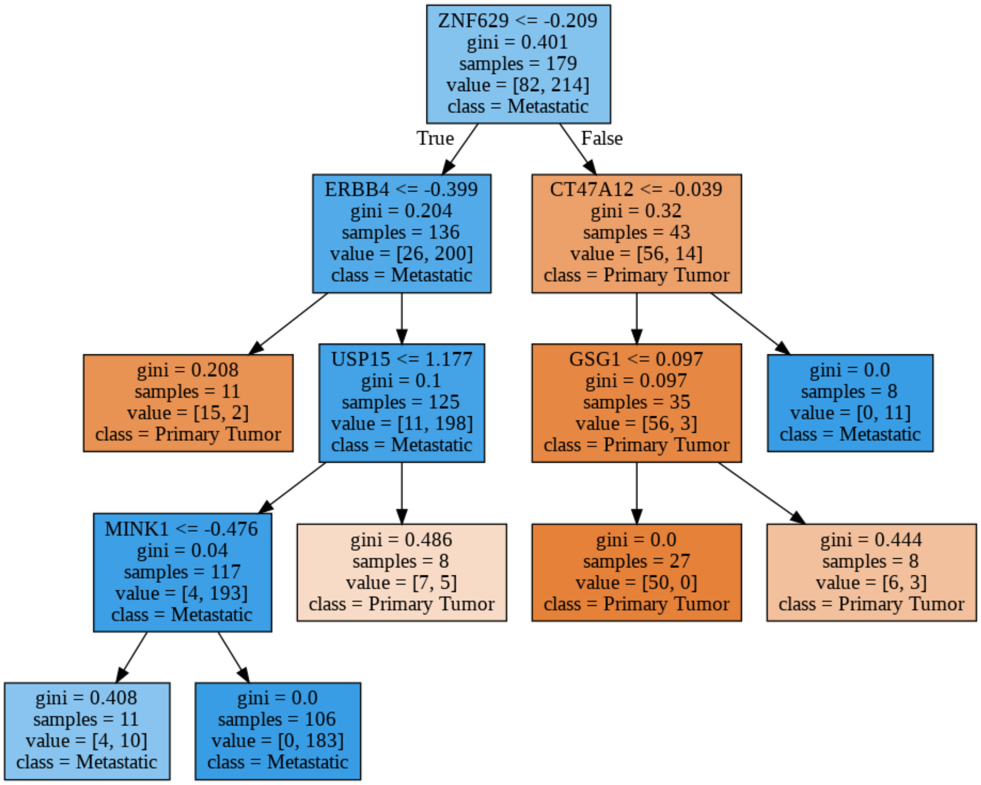

Figure S 6. Biomarker Discovery - Random Forest Estimator 2

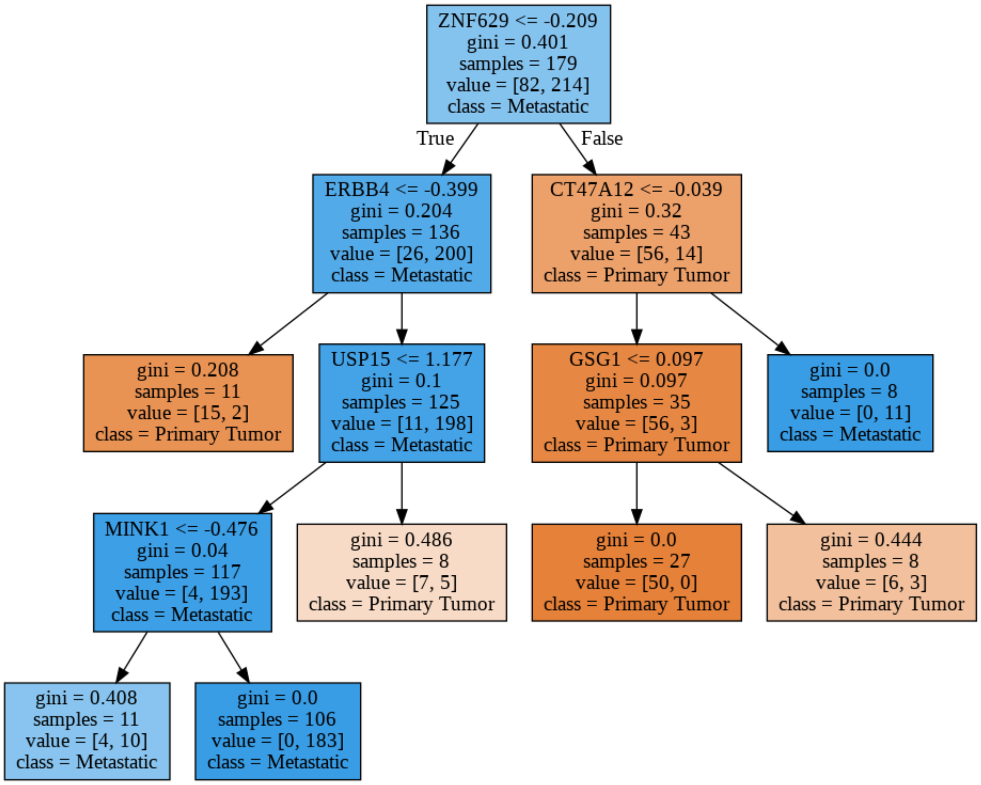

Figure S 7. Biomarker Discovery - Random Forest Estimator 3

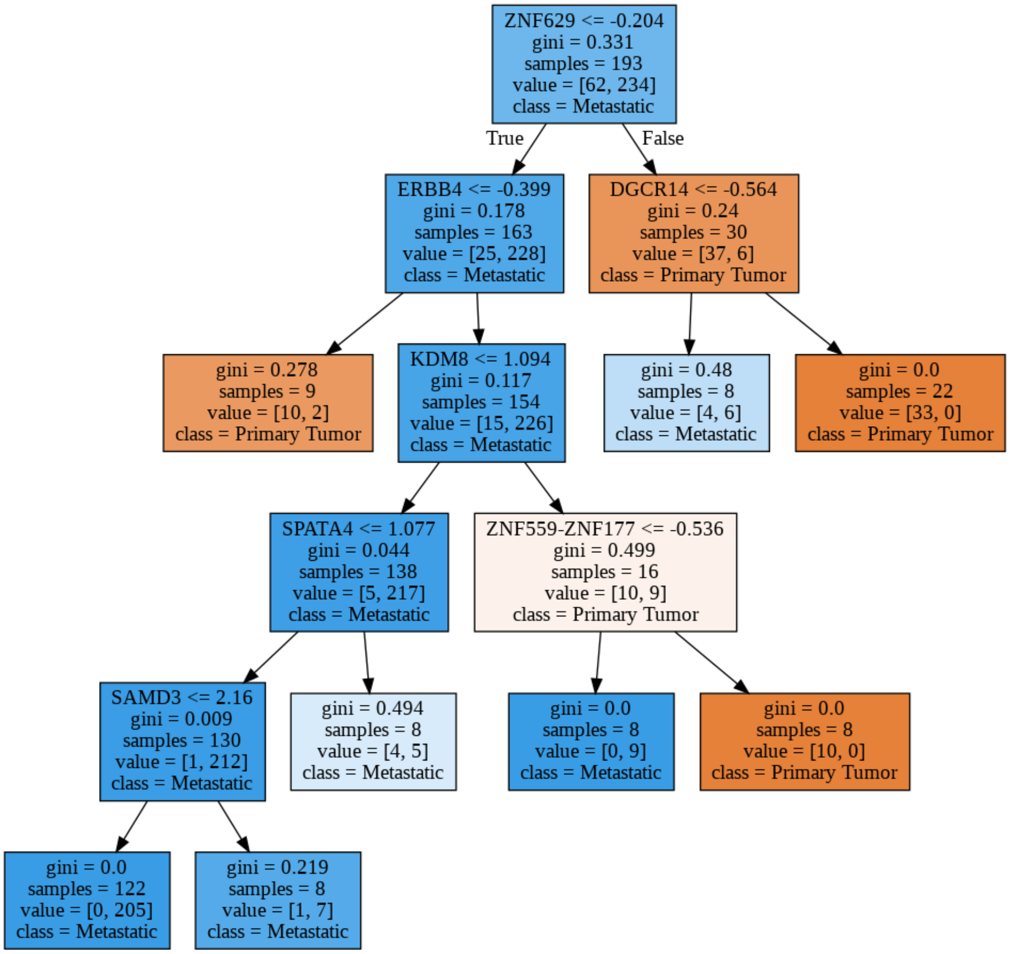

Figure S 8. Biomarker Discovery - Random Forest Estimator 4

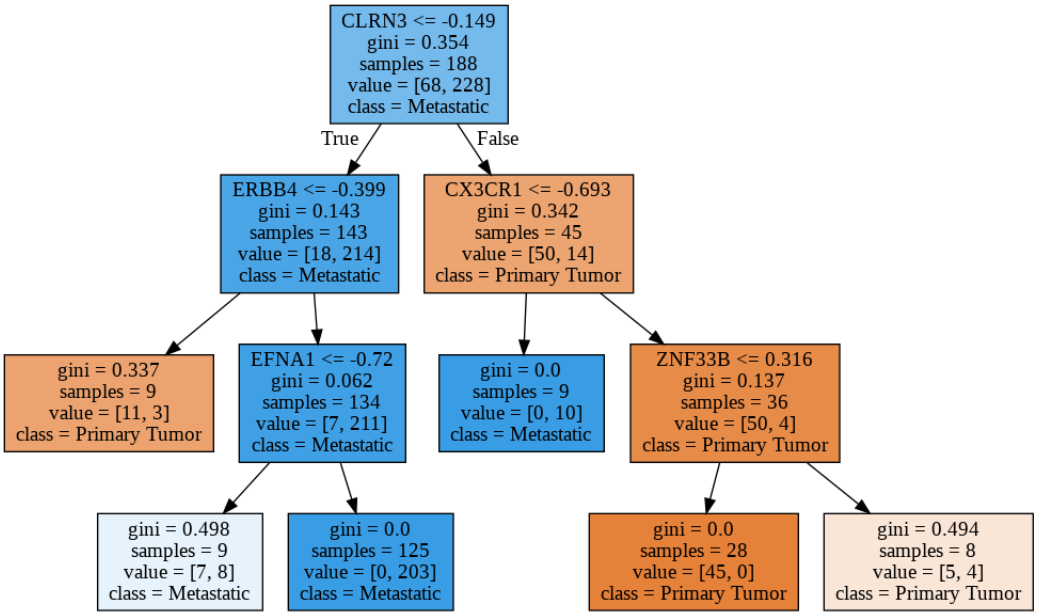

Figure S 9. Biomarker Discovery - Random Forest Estimator 5

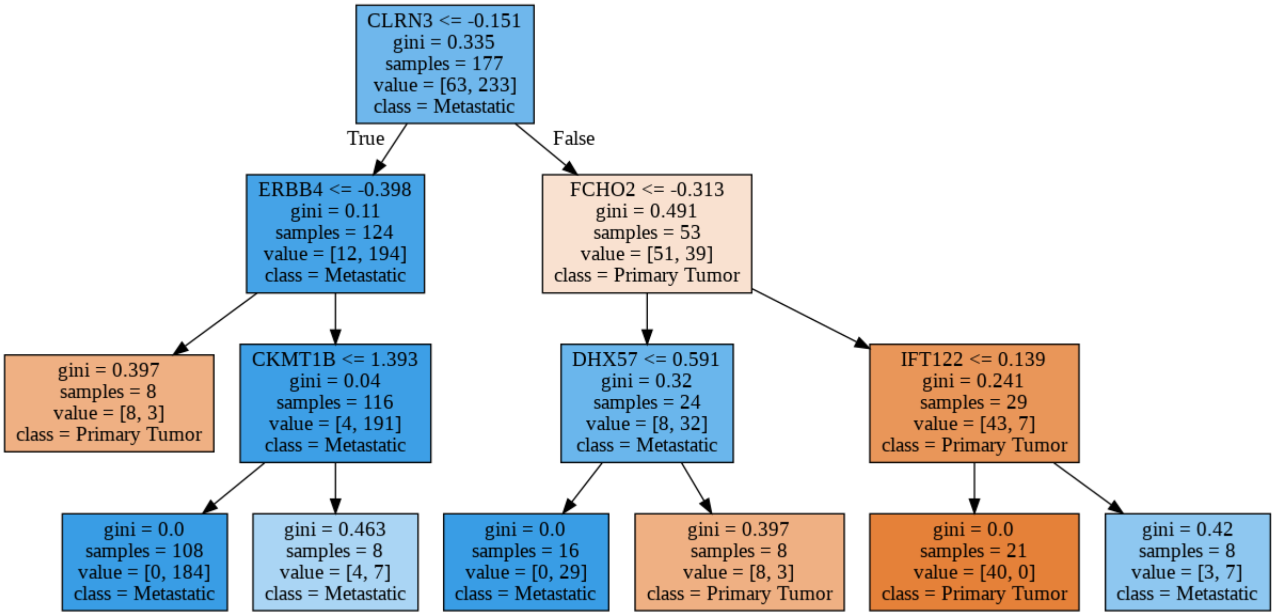

Figure S 10. Biomarker Discovery - Random Forest Estimator 6

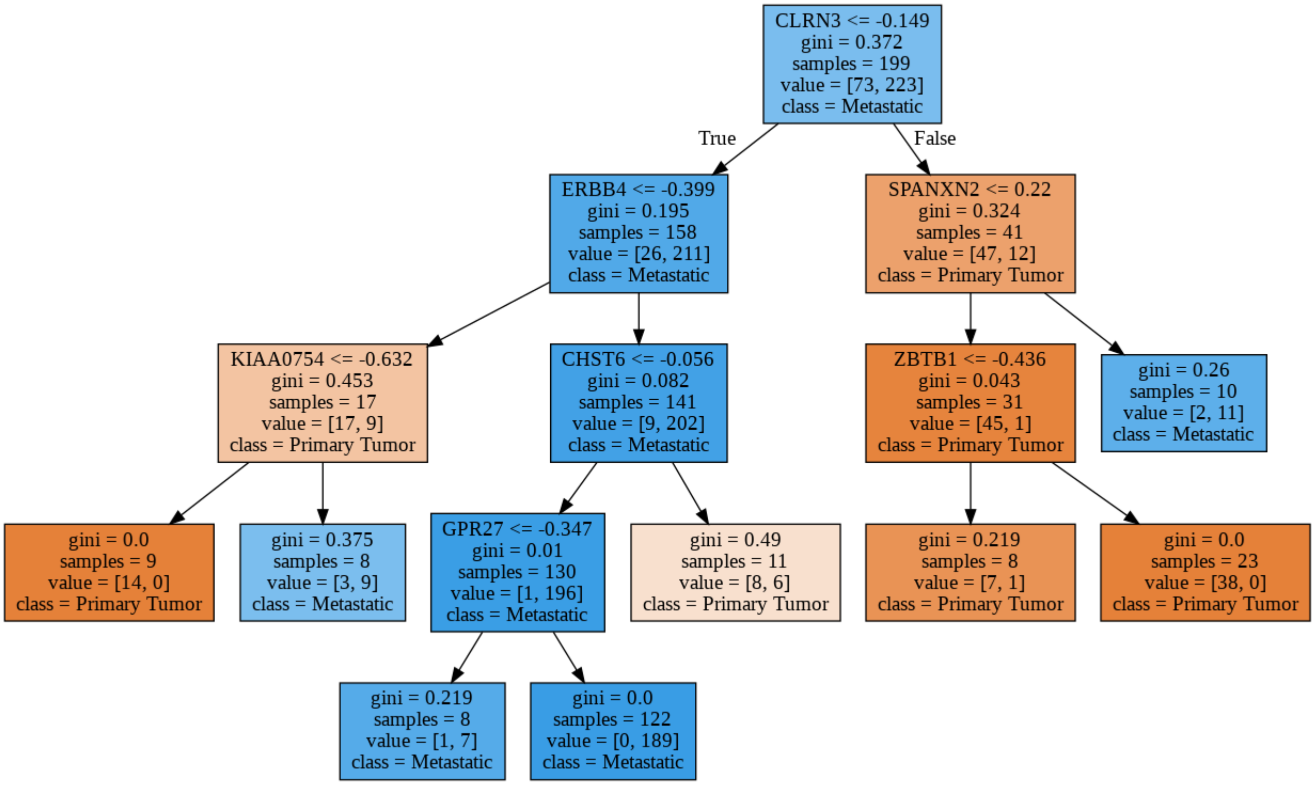

Figure S 11. Biomarker Discovery - Random Forest Estimator 7

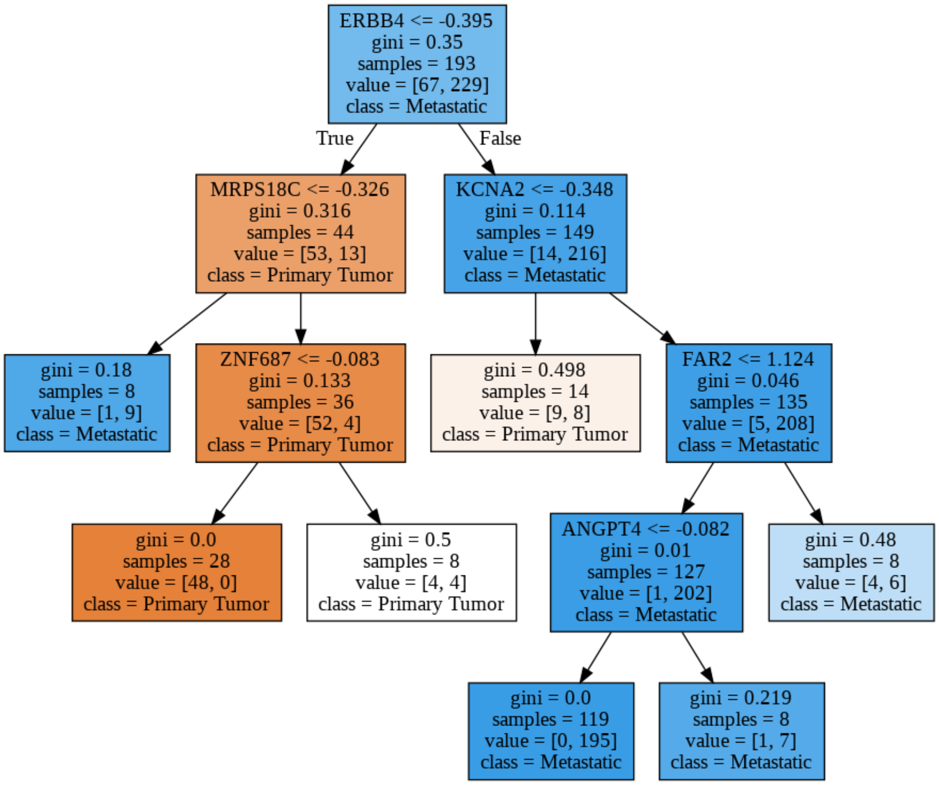

Figure S 12. Biomarker Discovery - Random Forest Estimator 8

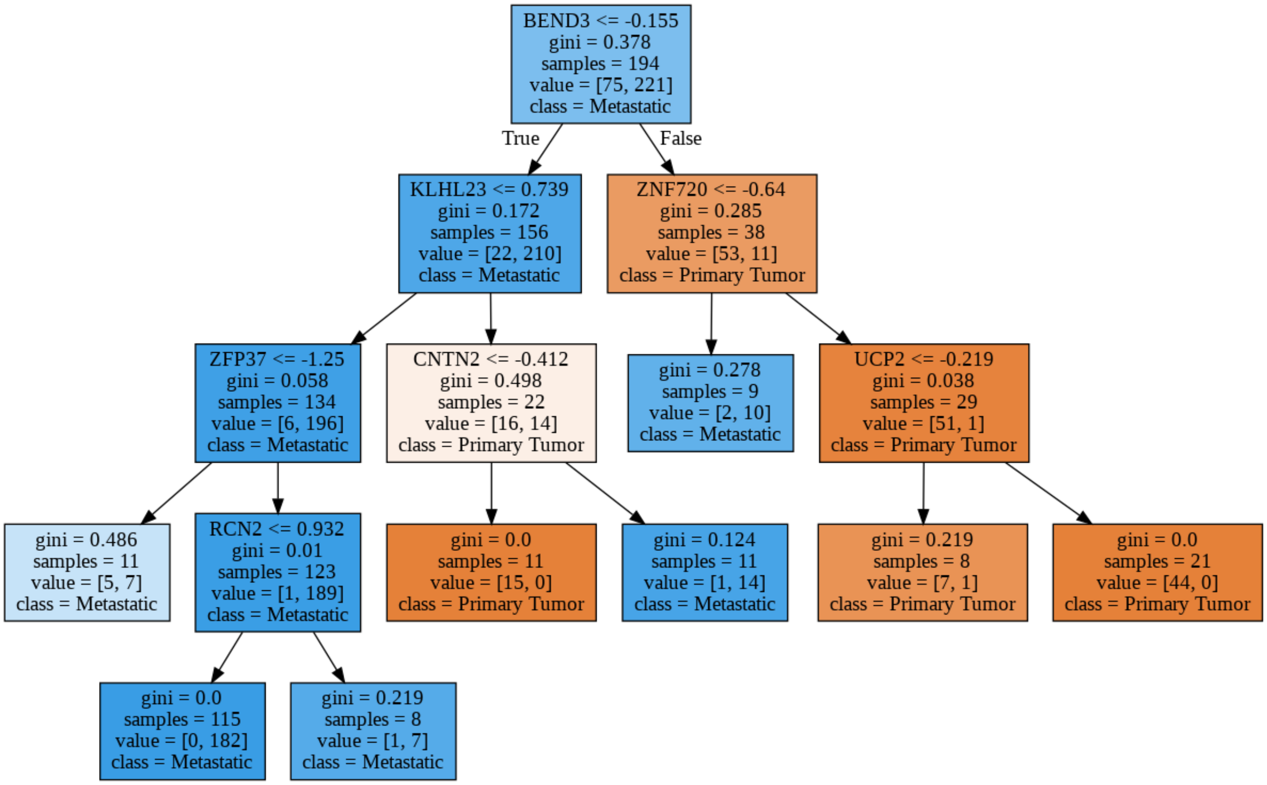

Figure S 13. Biomarker Discovery - Random Forest Estimator 9

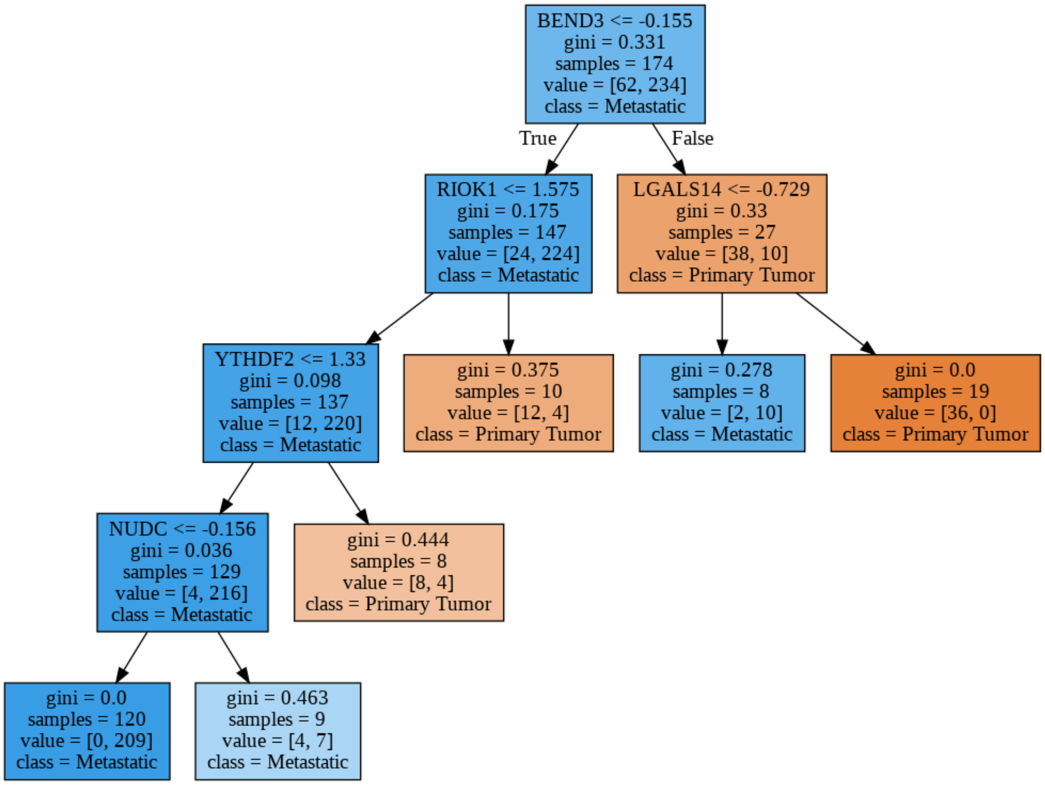

Figure S 14. Biomarker Discovery - Random Forest Estimator 10

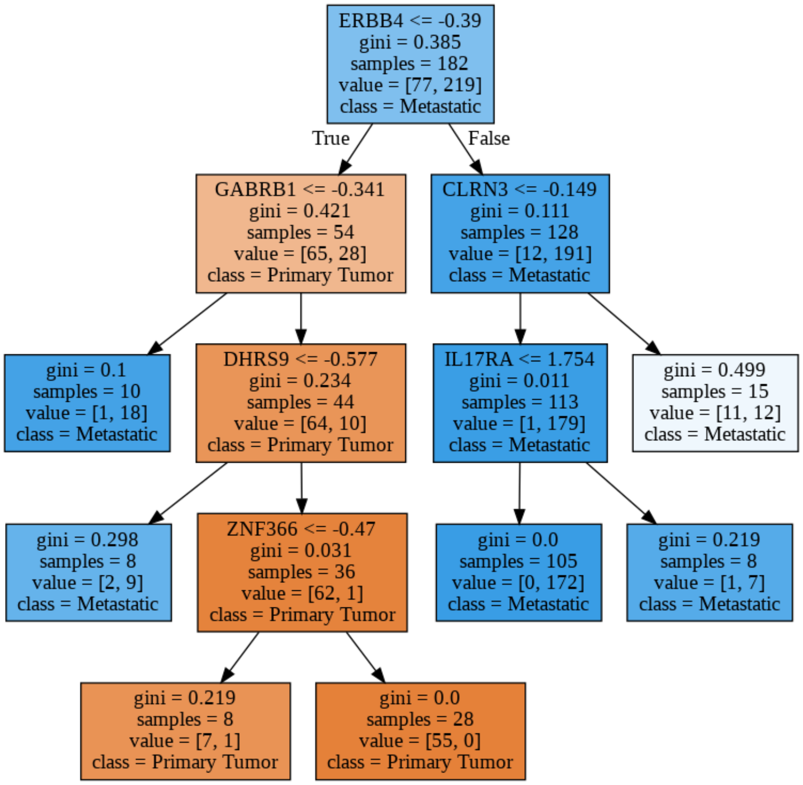

Figure S 15. Biomarker Discovery - Random Forest Estimator 11

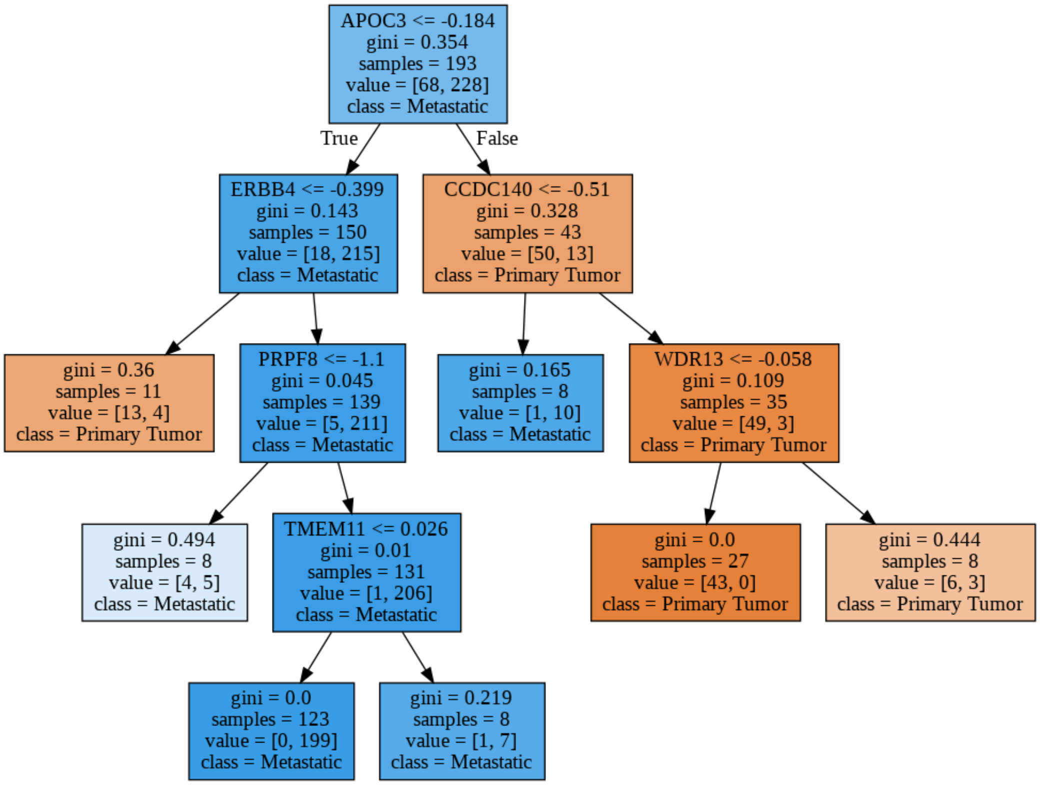

Figure S 16. Biomarker Discovery - Random Forest Estimator 12

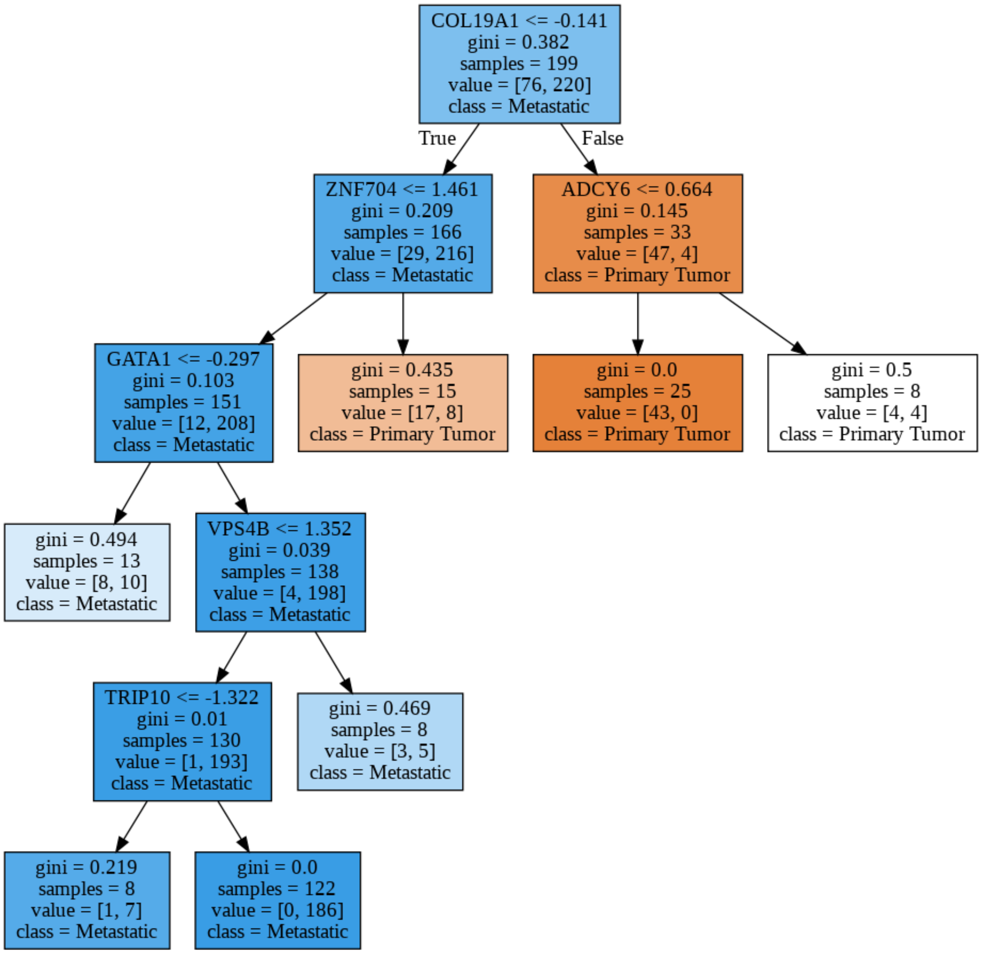

Figure S 17. Biomarker Discovery - Random Forest Estimator 13

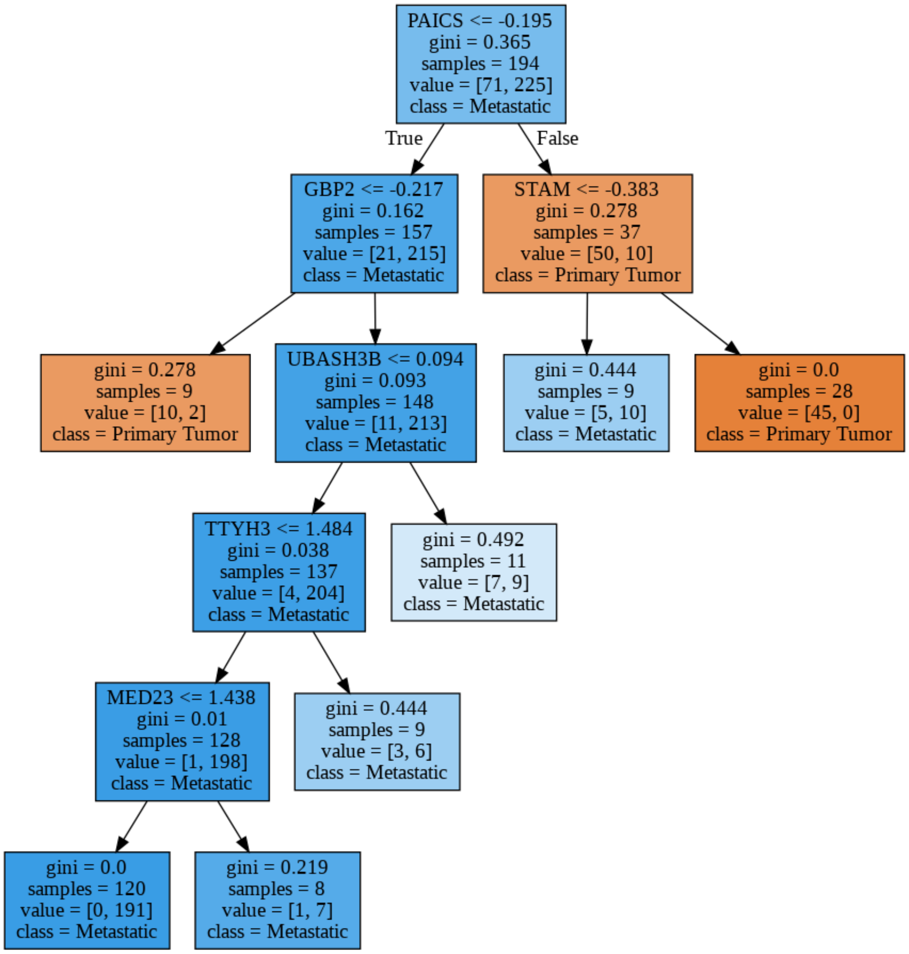

Figure S 18. Biomarker Discovery - Random Forest Estimator 14

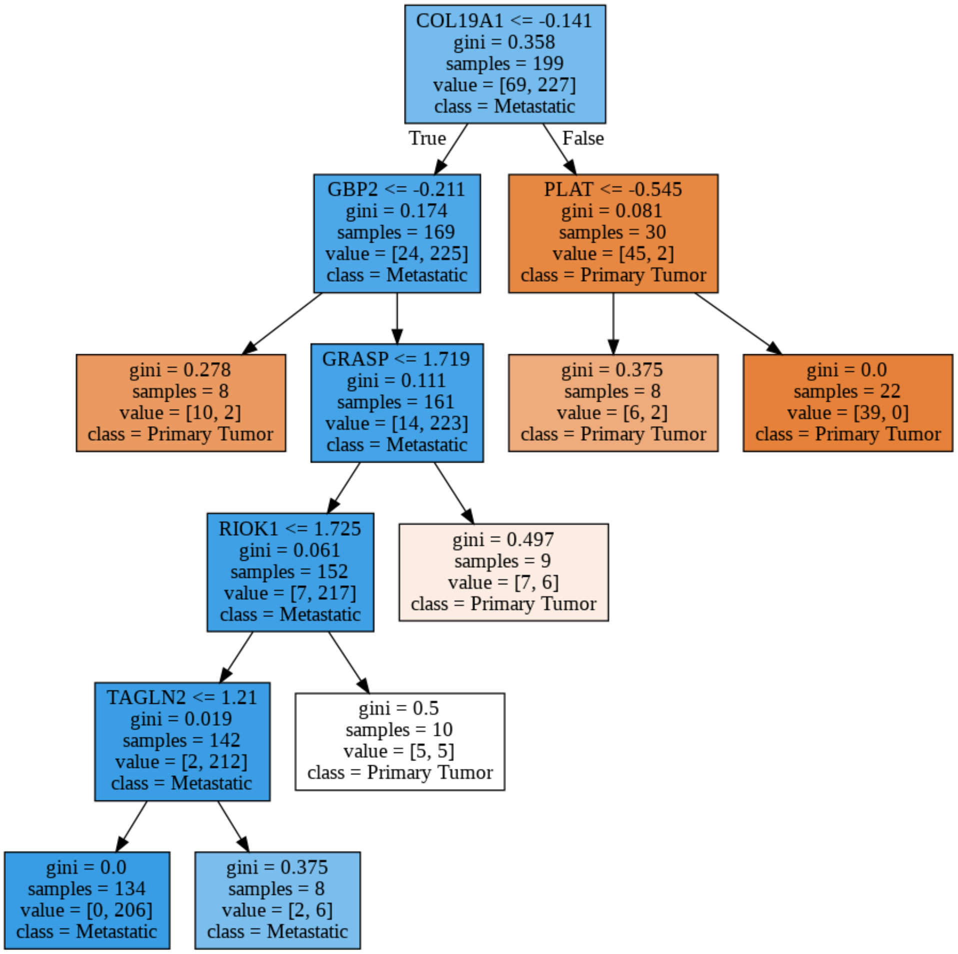

Figure S 19. Biomarker Discovery - Random Forest Estimator 15

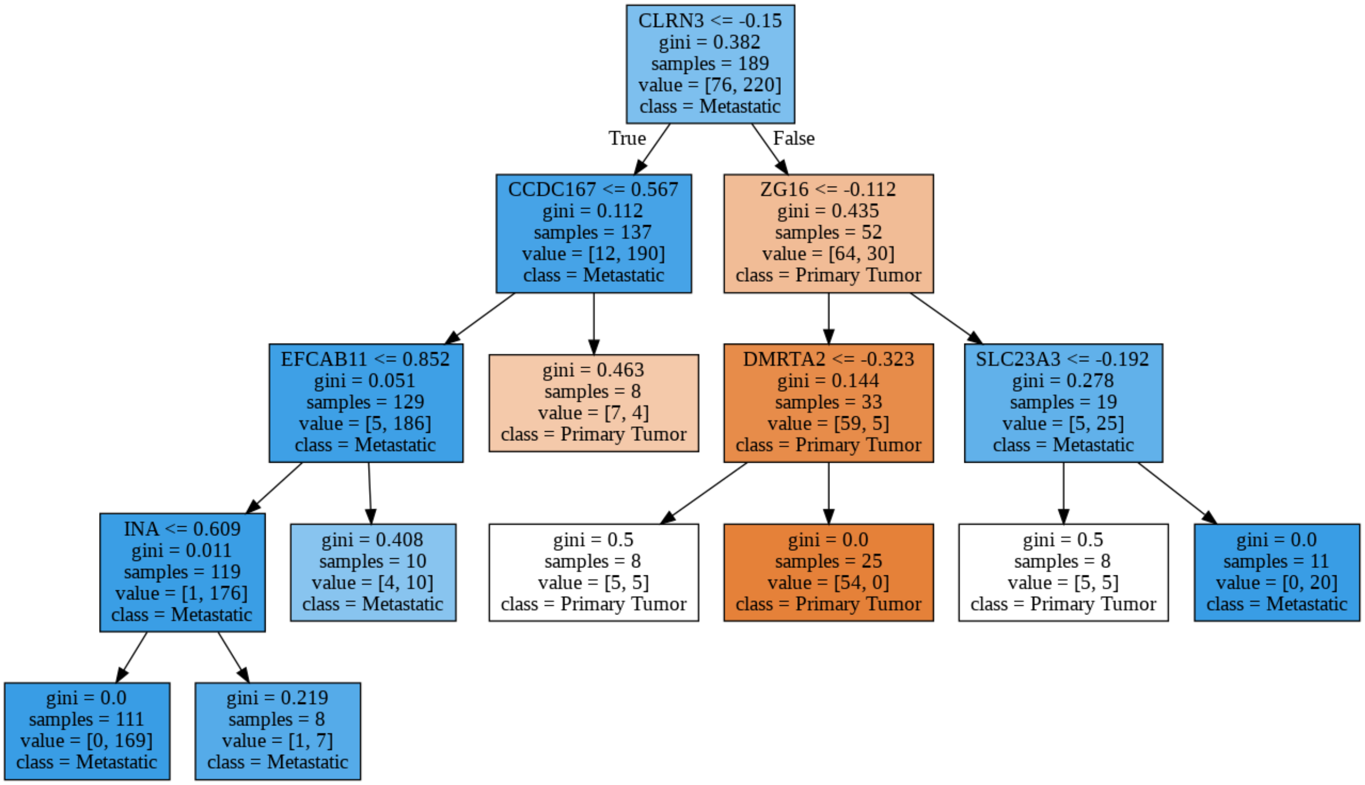

Figure S 20. Biomarker Discovery - Random Forest Estimator 16

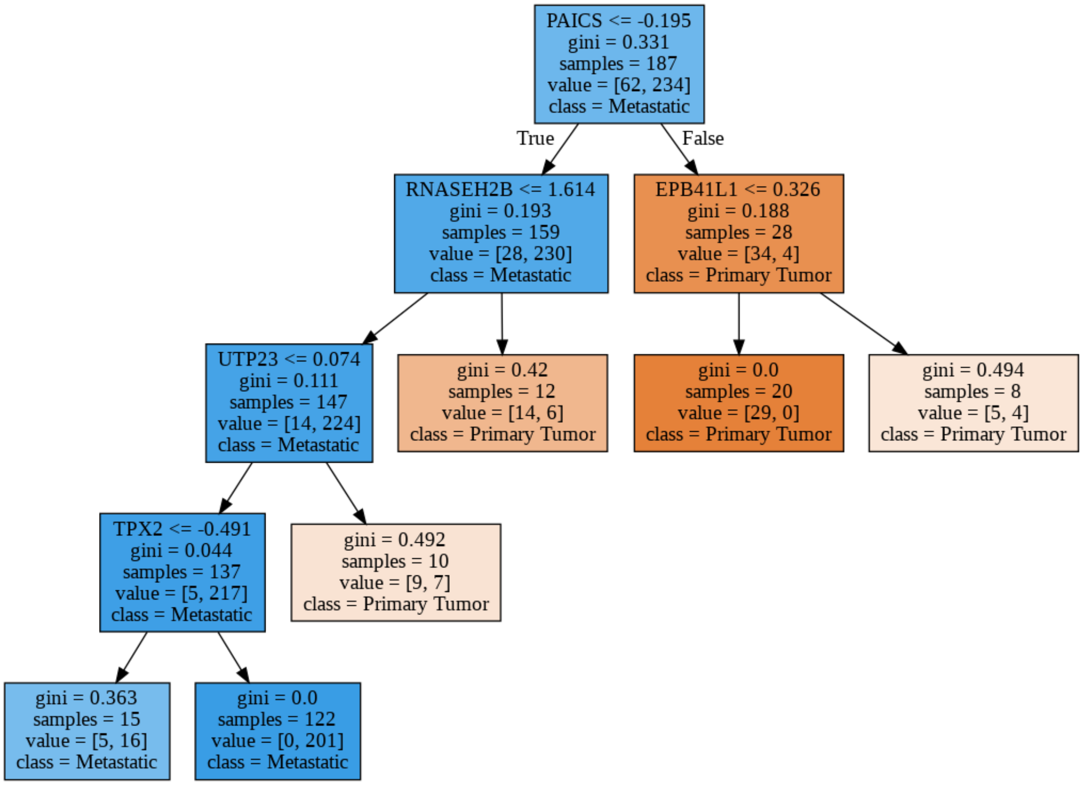

Figure S 21. Biomarker Discovery - Random Forest Estimator 17

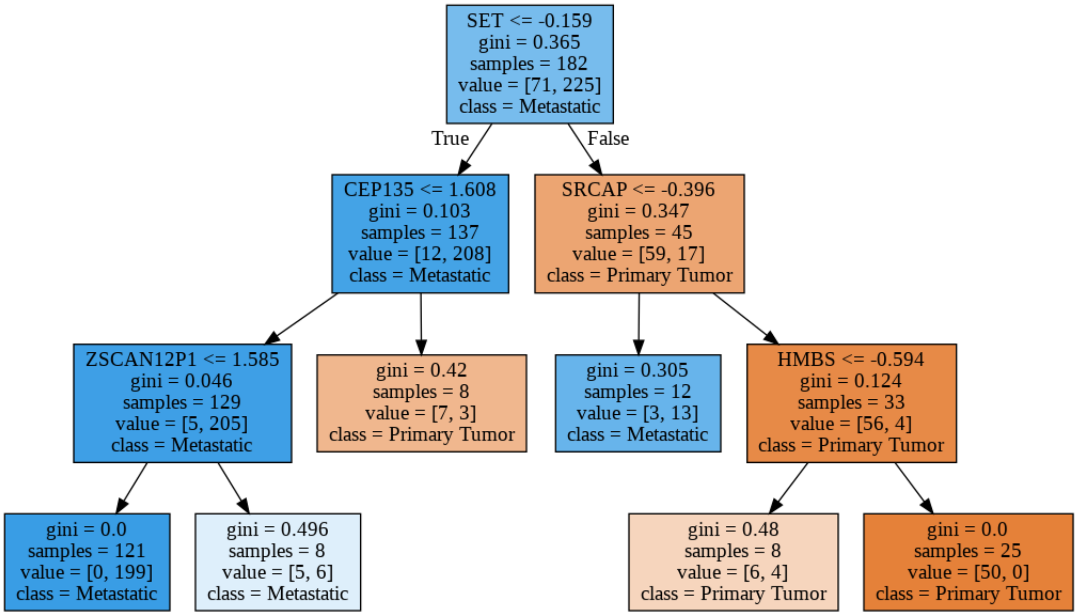

Figure S 22. Biomarker Discovery - Random Forest Estimator 18

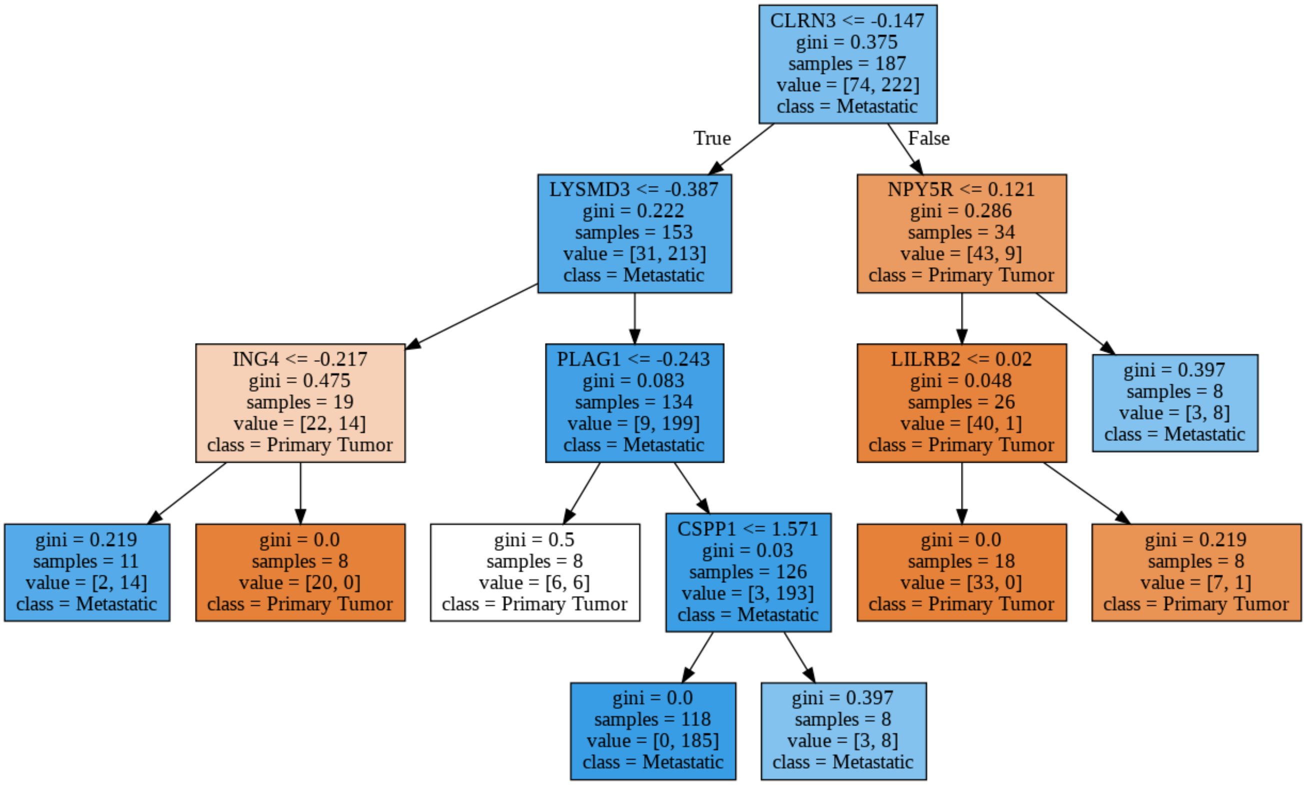

Figure S 23. Biomarker Discovery - Random Forest Estimator 19

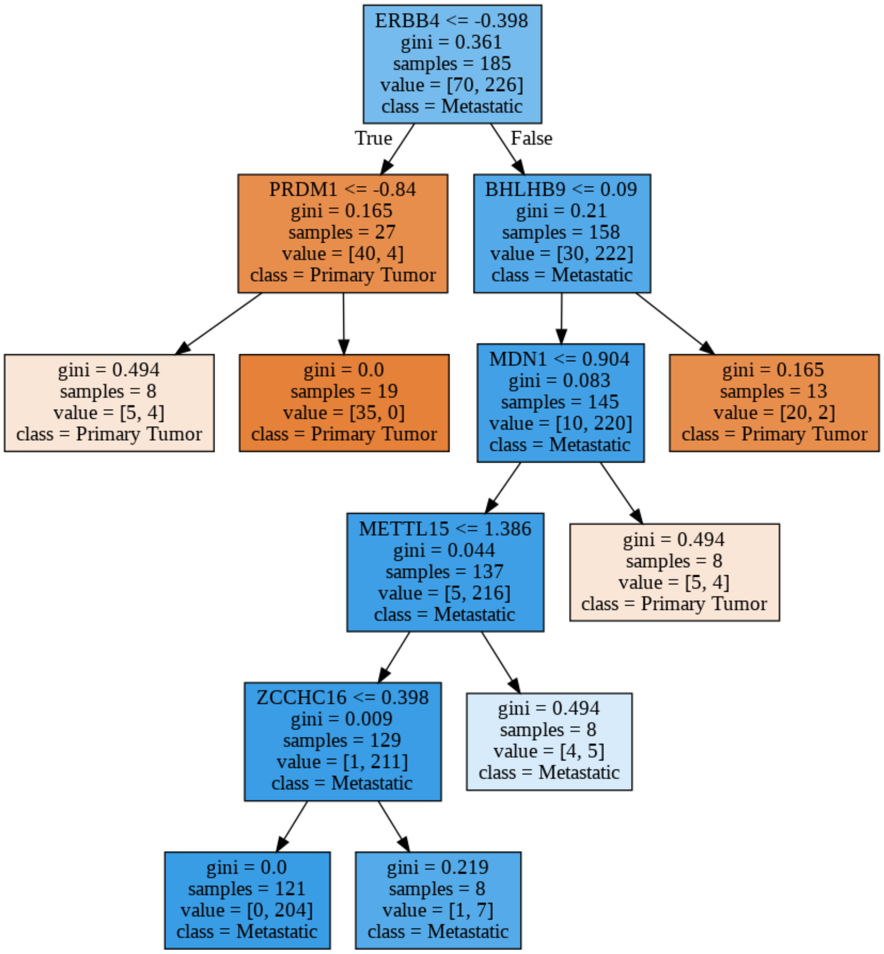

Figure S 24. Biomarker Discovery - Random Forest Estimator 20

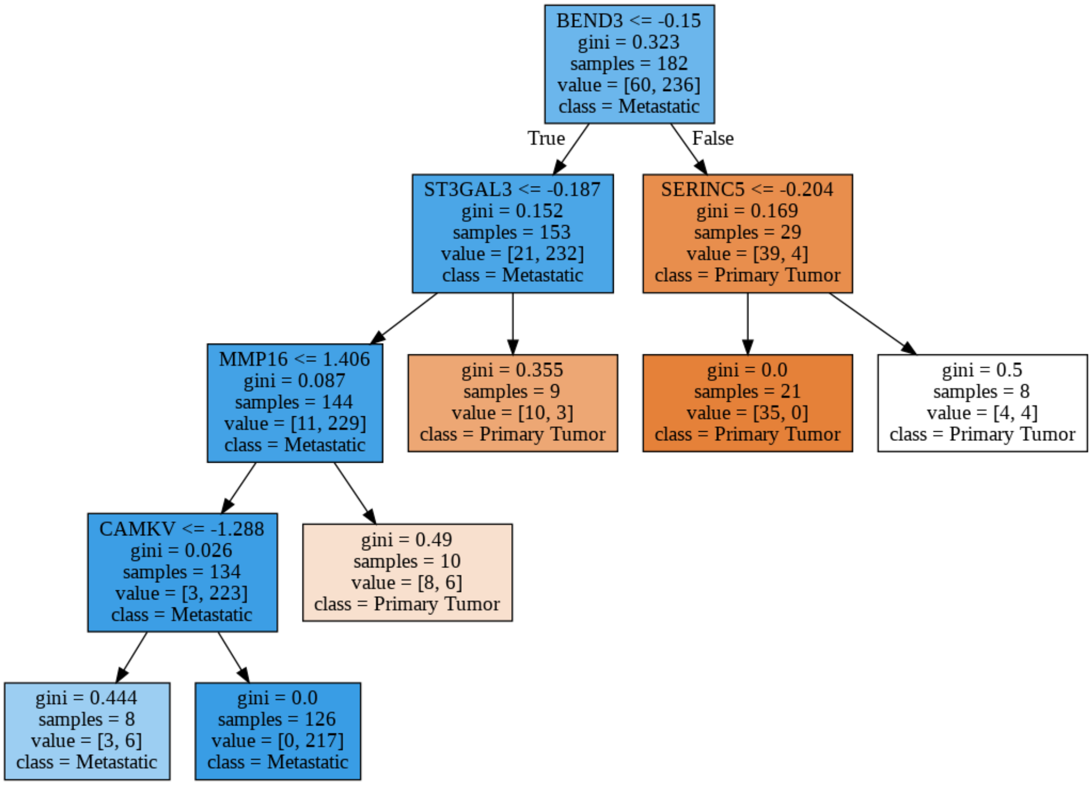

Figure S 25. Biomarker Discovery - Random Forest Estimator 21

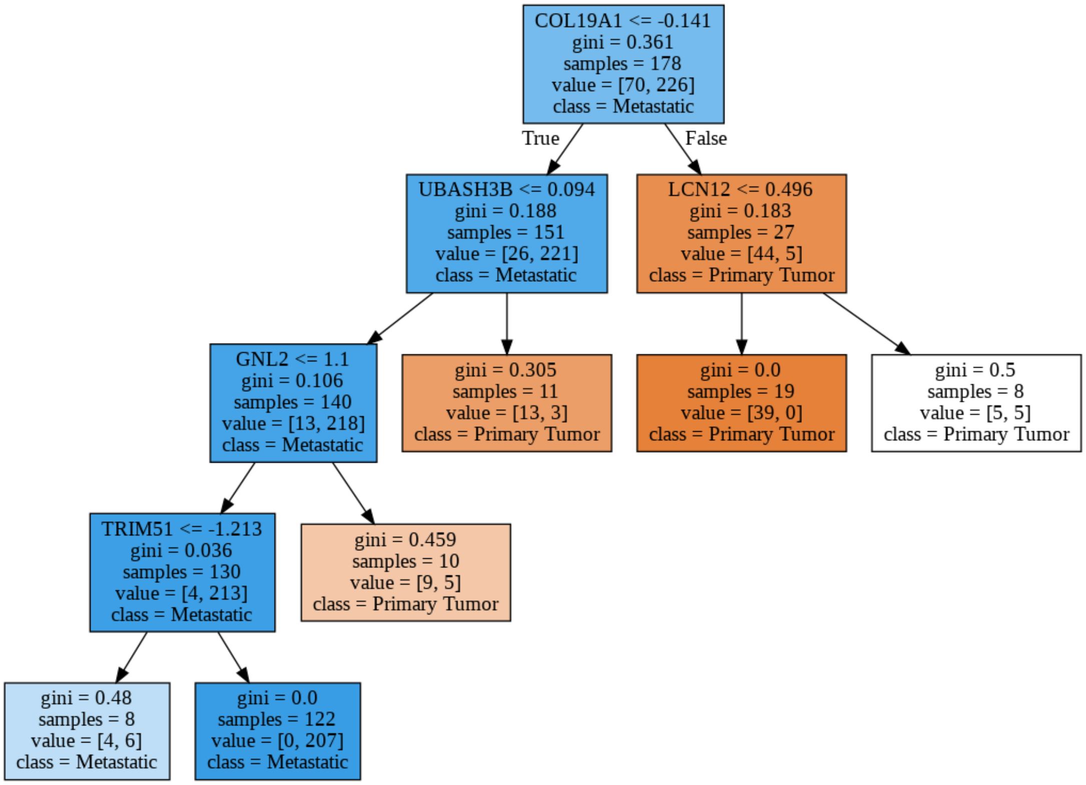

Figure S 26. Biomarker Discovery - Random Forest Estimator 22

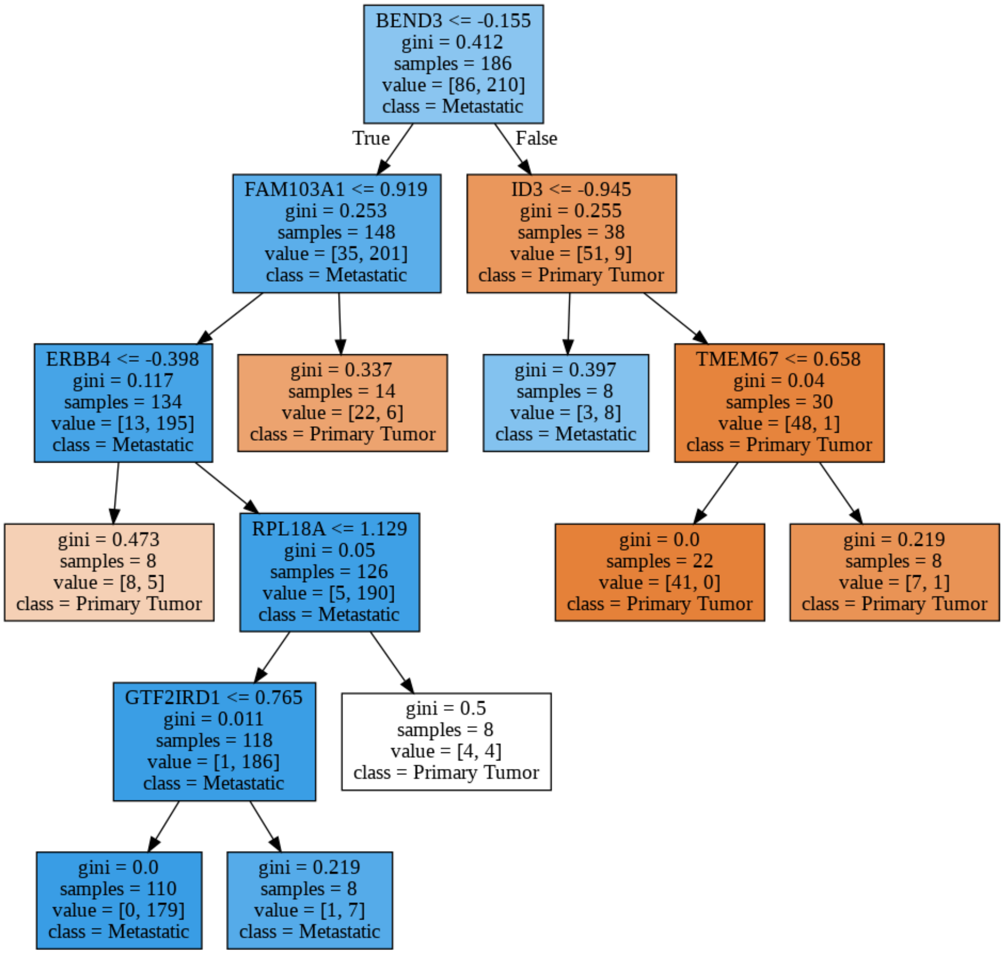

Figure S 27. Biomarker Discovery - Random Forest Estimator 23

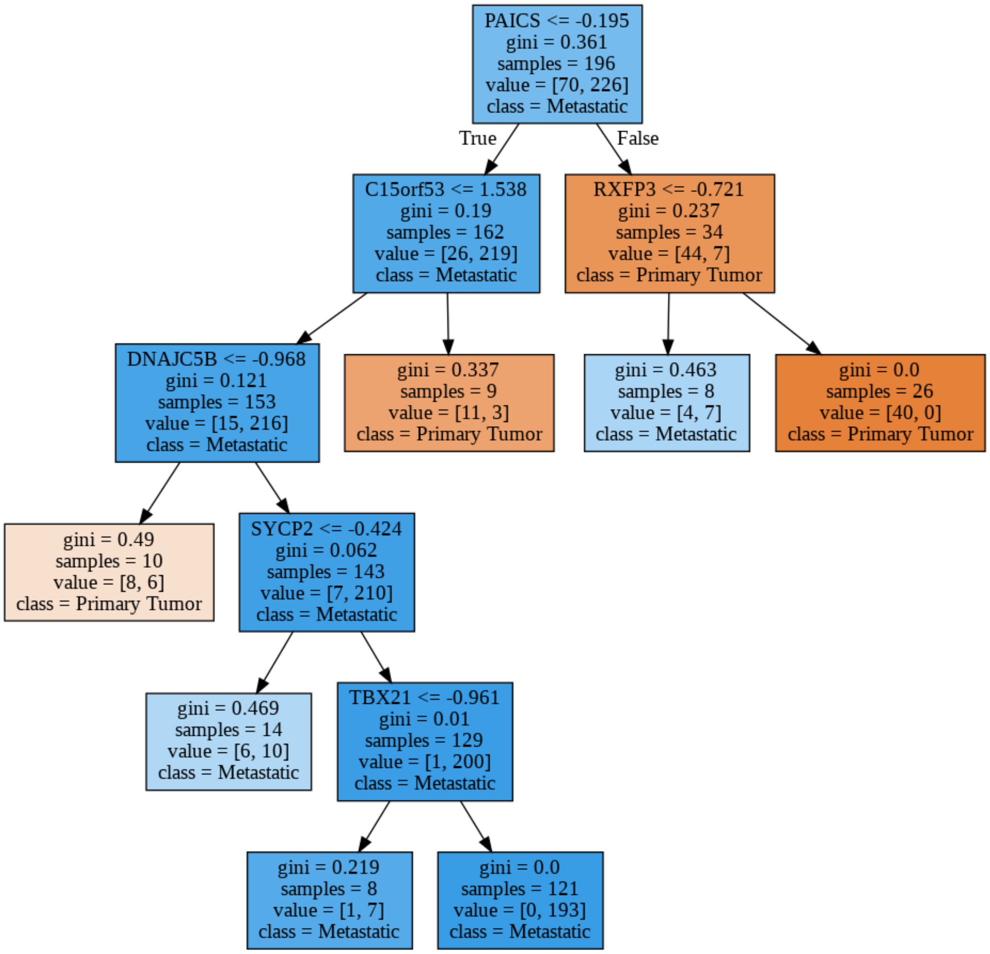

Figure S 28. Biomarker Discovery - Random Forest Estimator 24

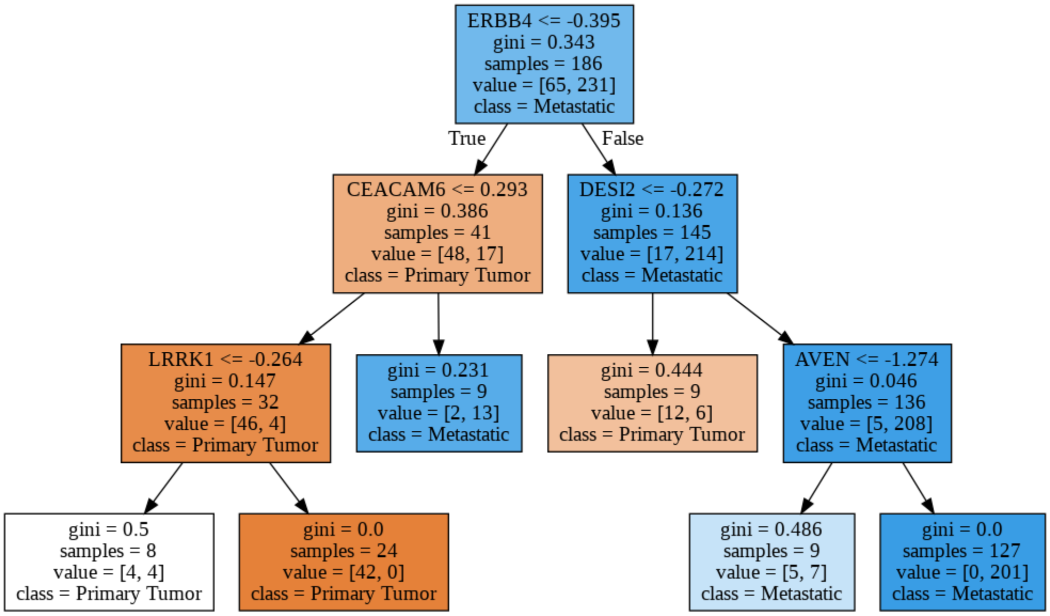

Figure S 29. Biomarker Discovery - Random Forest Estimator 25

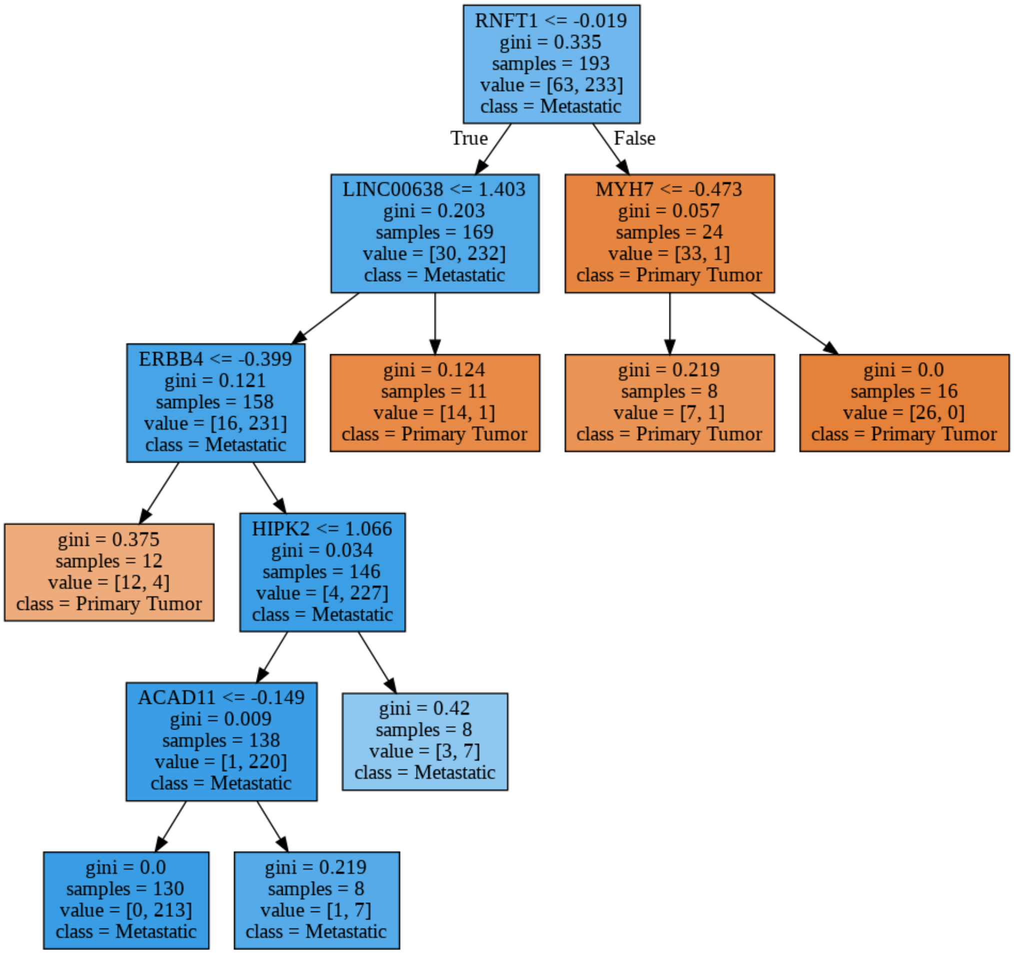

Figure S 30. Biomarker Discovery - Random Forest Estimator 26

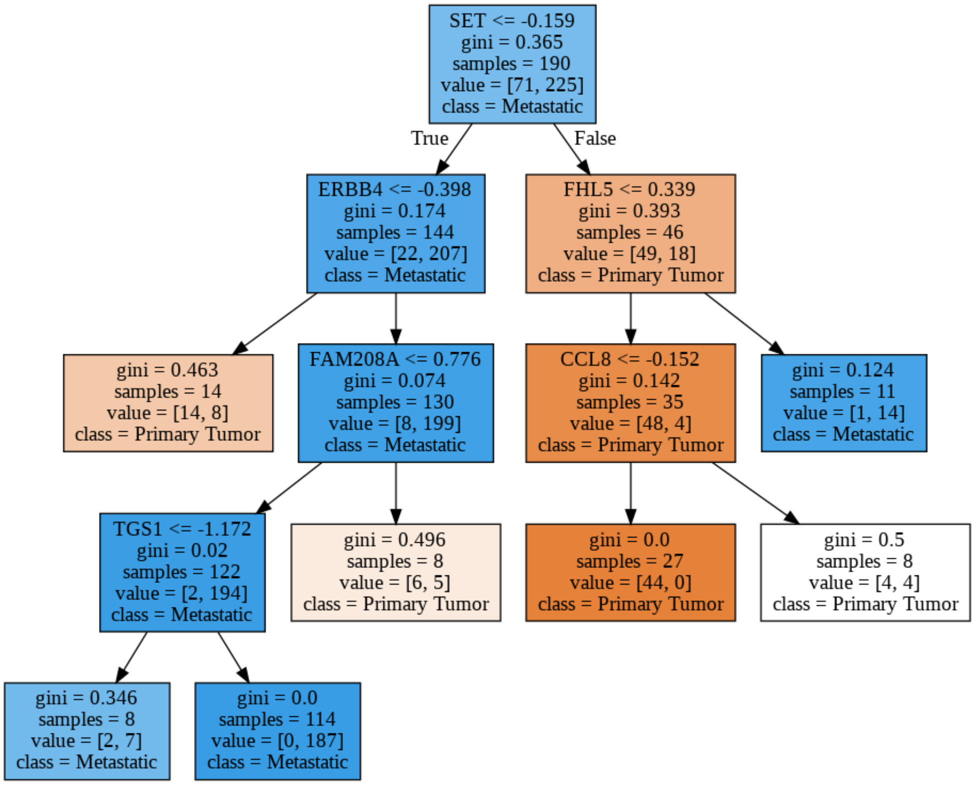

Figure S 31. Biomarker Discovery - Random Forest Estimator 27

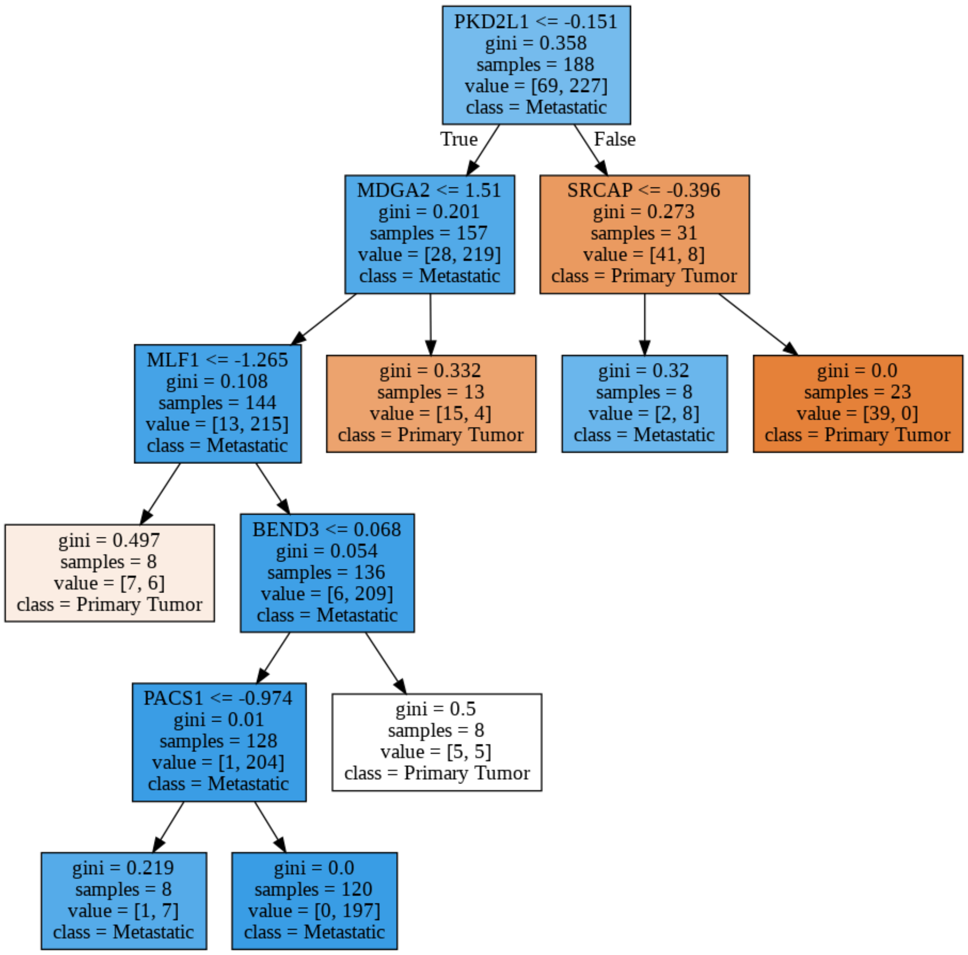

Figure S 32. Biomarker Discovery - Random Forest Estimator 28

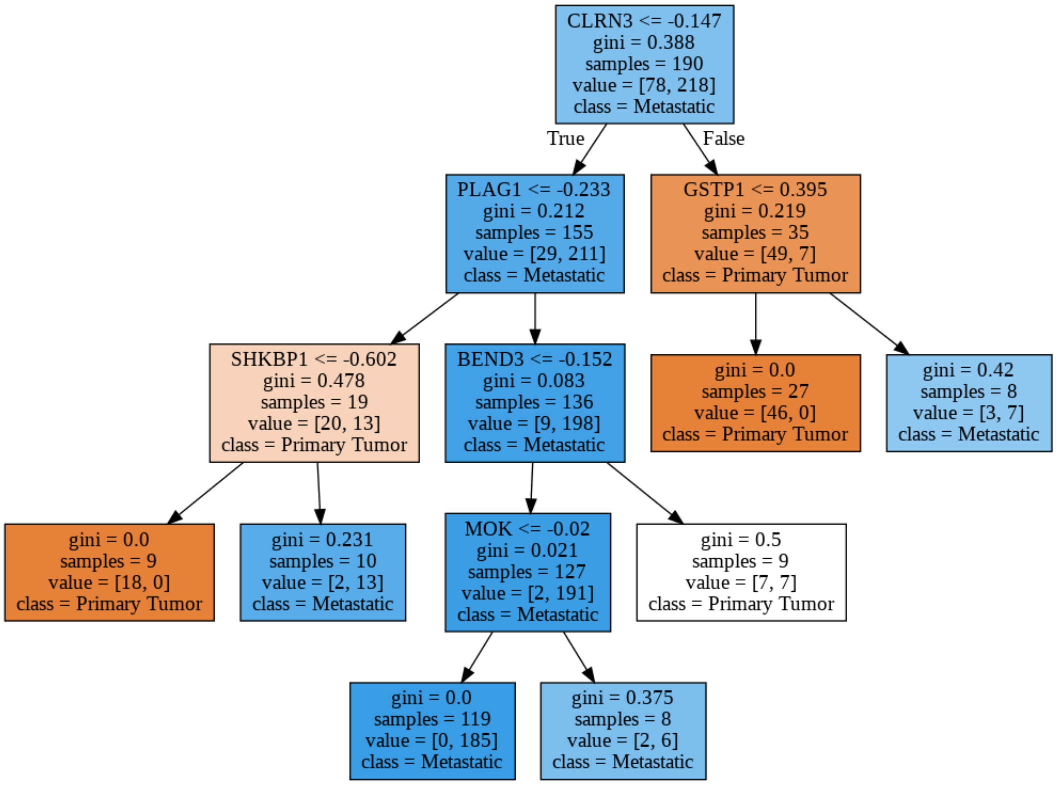

Figure S 33. Biomarker Discovery - Random Forest Estimator 29

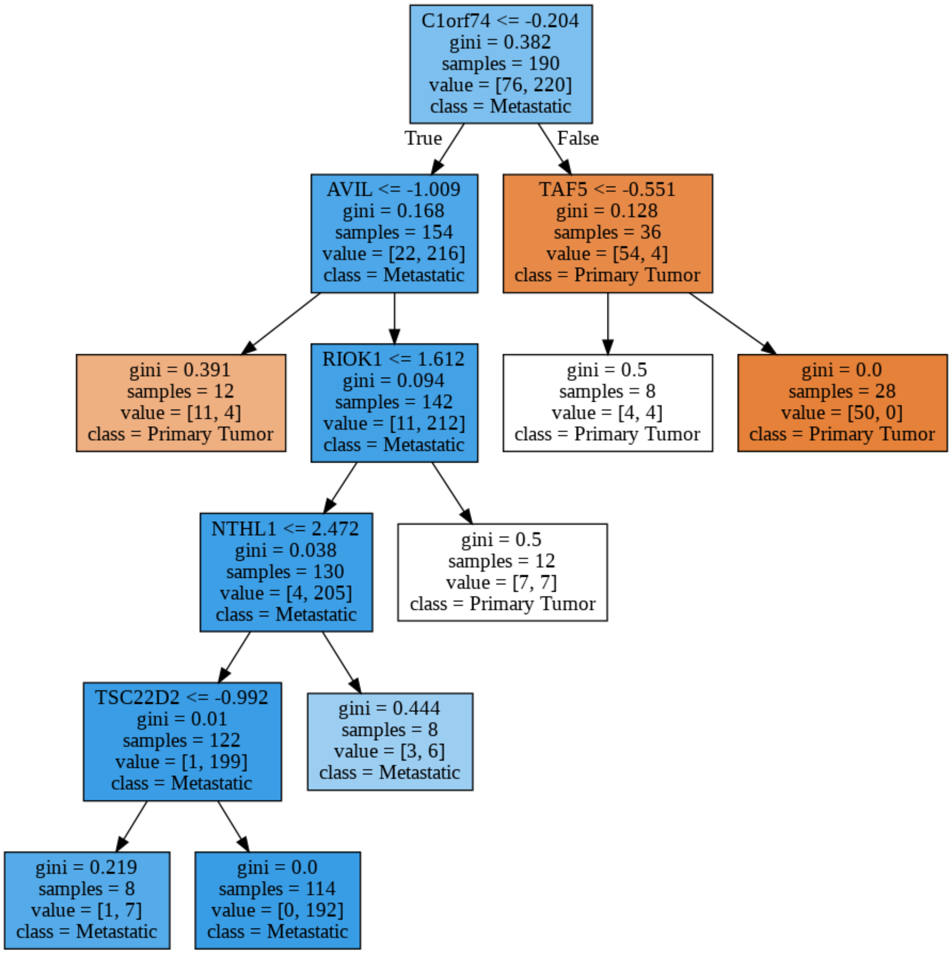

Figure S 34. Biomarker Discovery - Random Forest Estimator 30

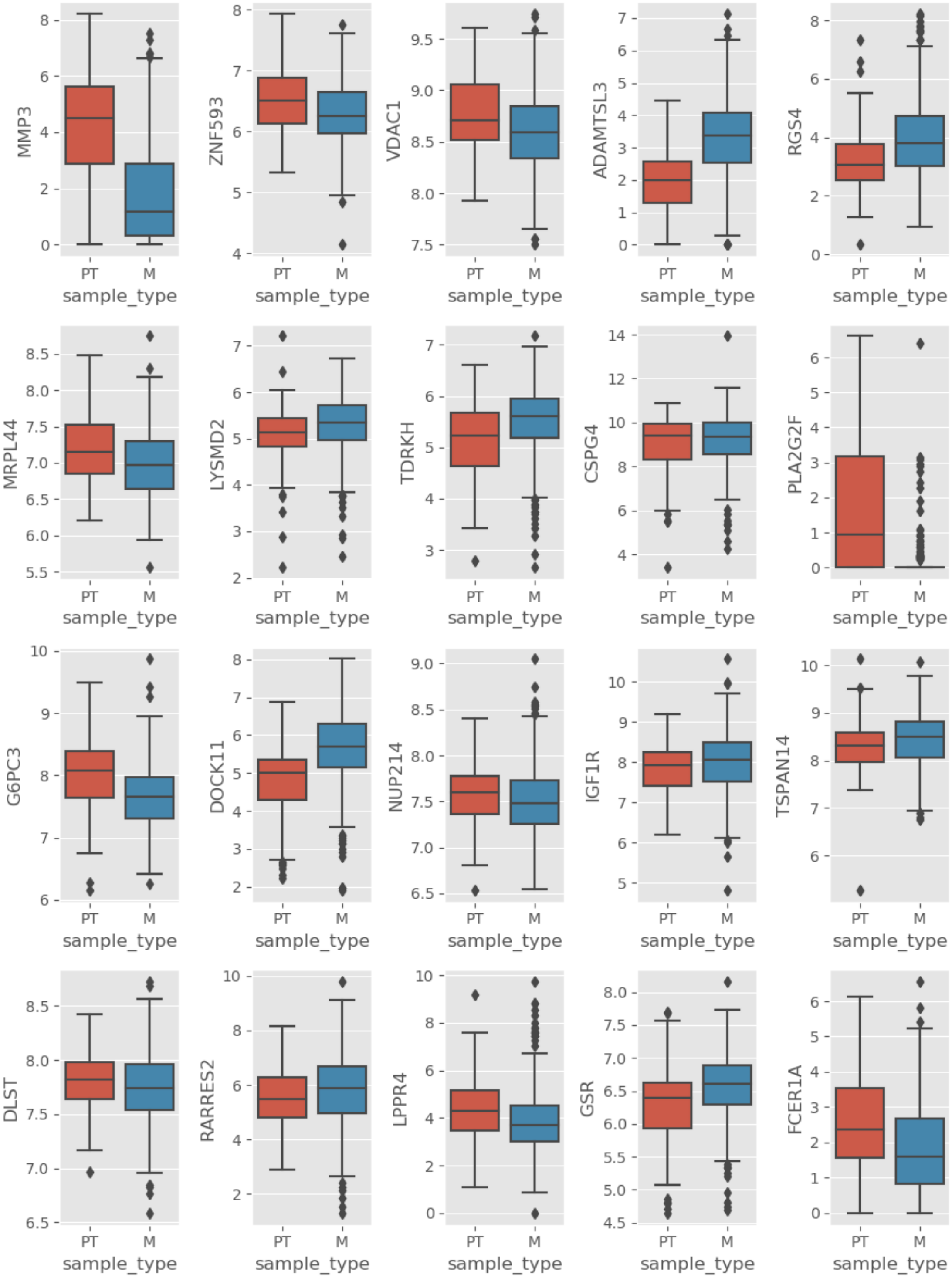

Figure S 35. Box Plots of Top 21 - 40 Gene Expressions.

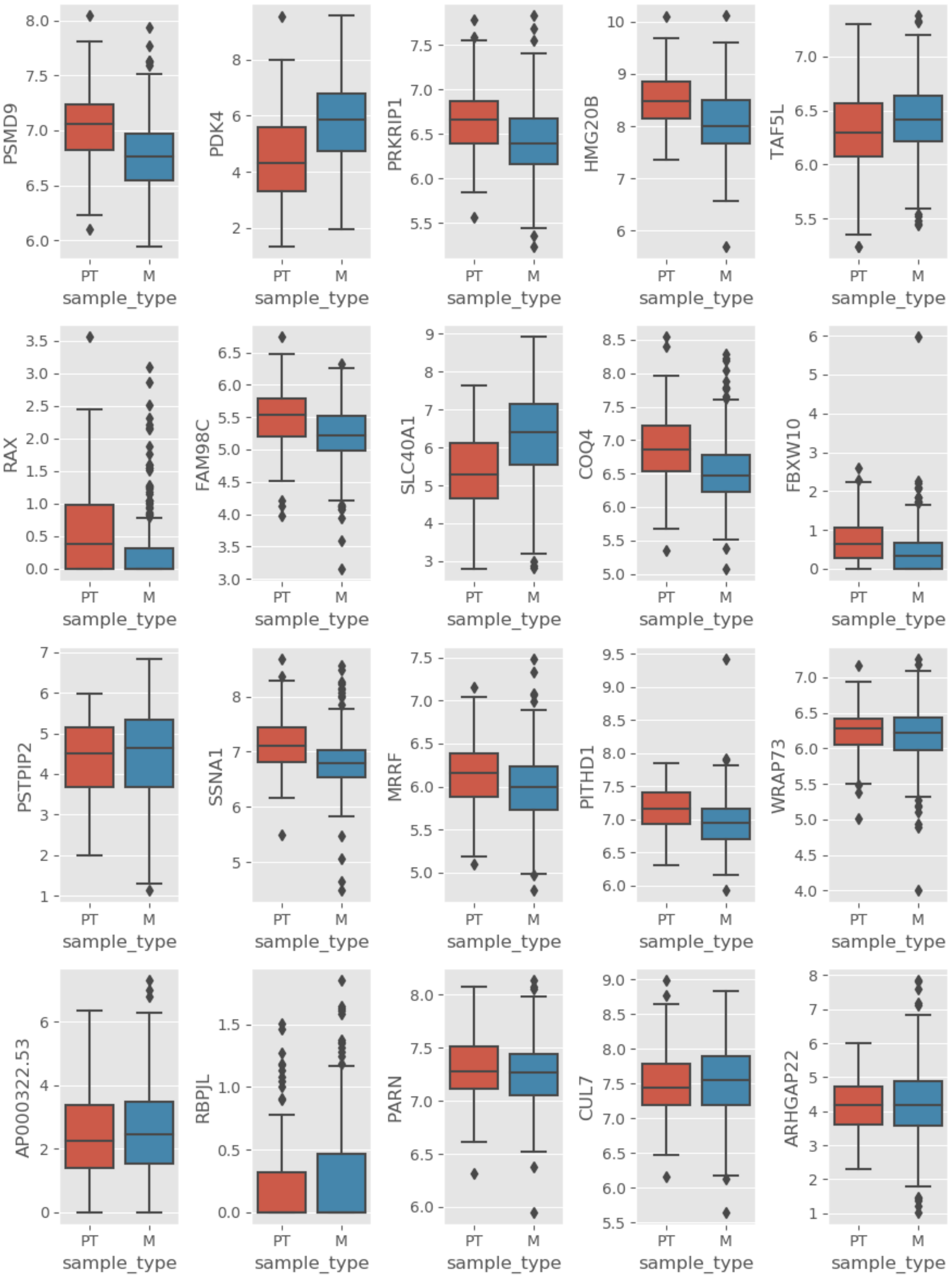

Figure S 36. Box Plots of Top 41 - 60 Gene Expressions.

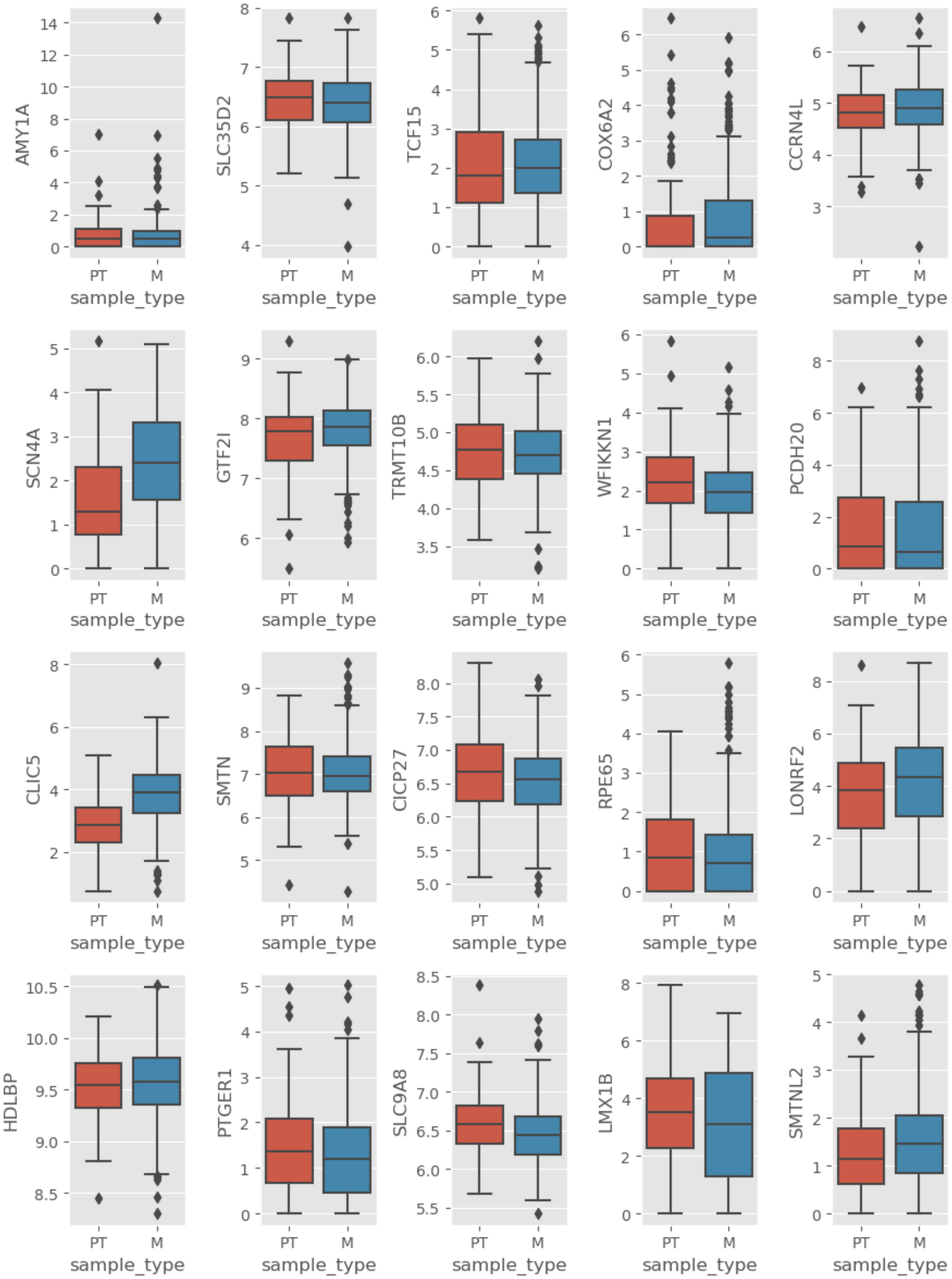

Figure S 37. Box Plots of Top 61 - 80 Gene Expressions.

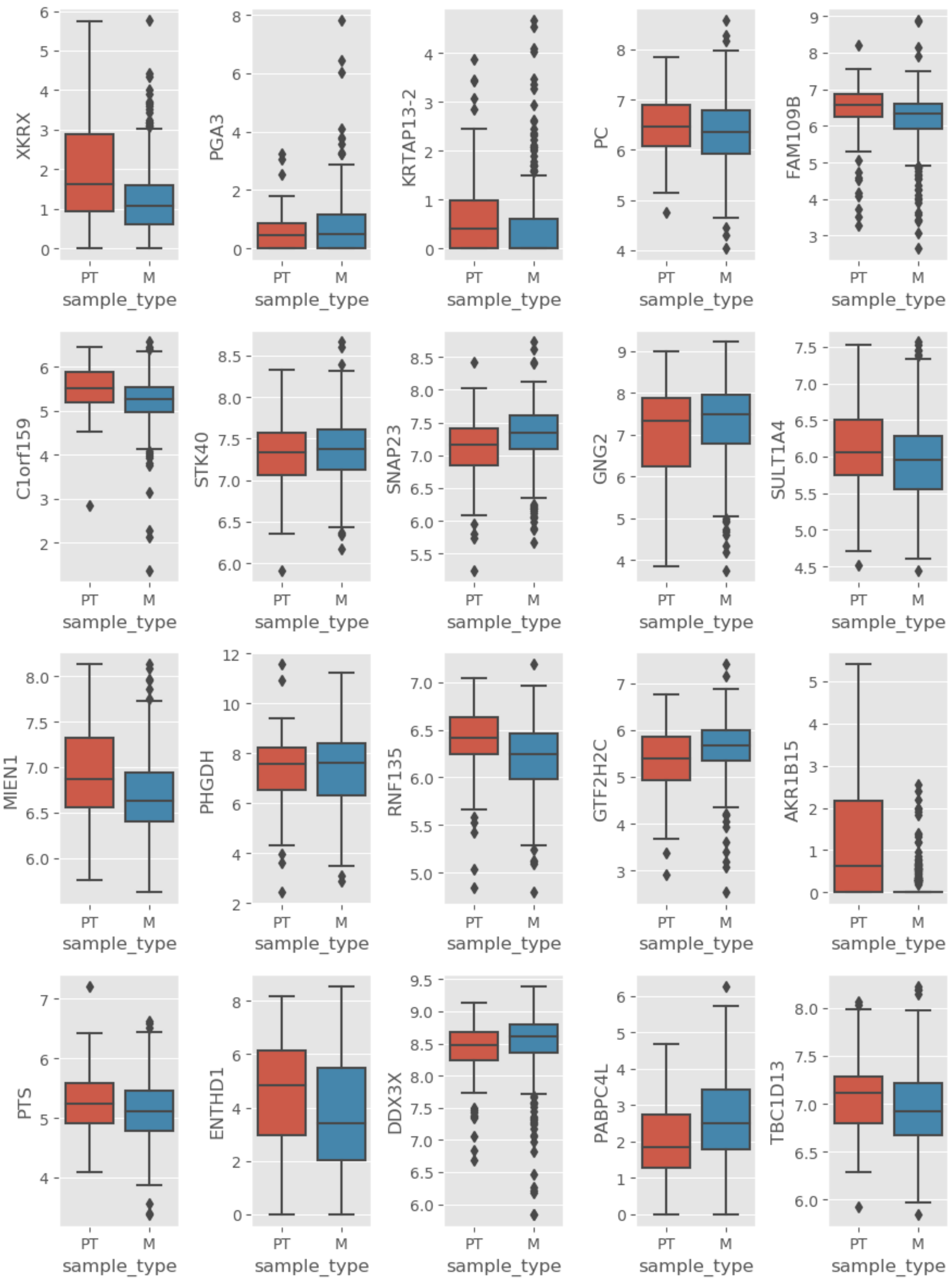

Figure S 38. Box Plots of Top 81 - 100 Gene Expressions.

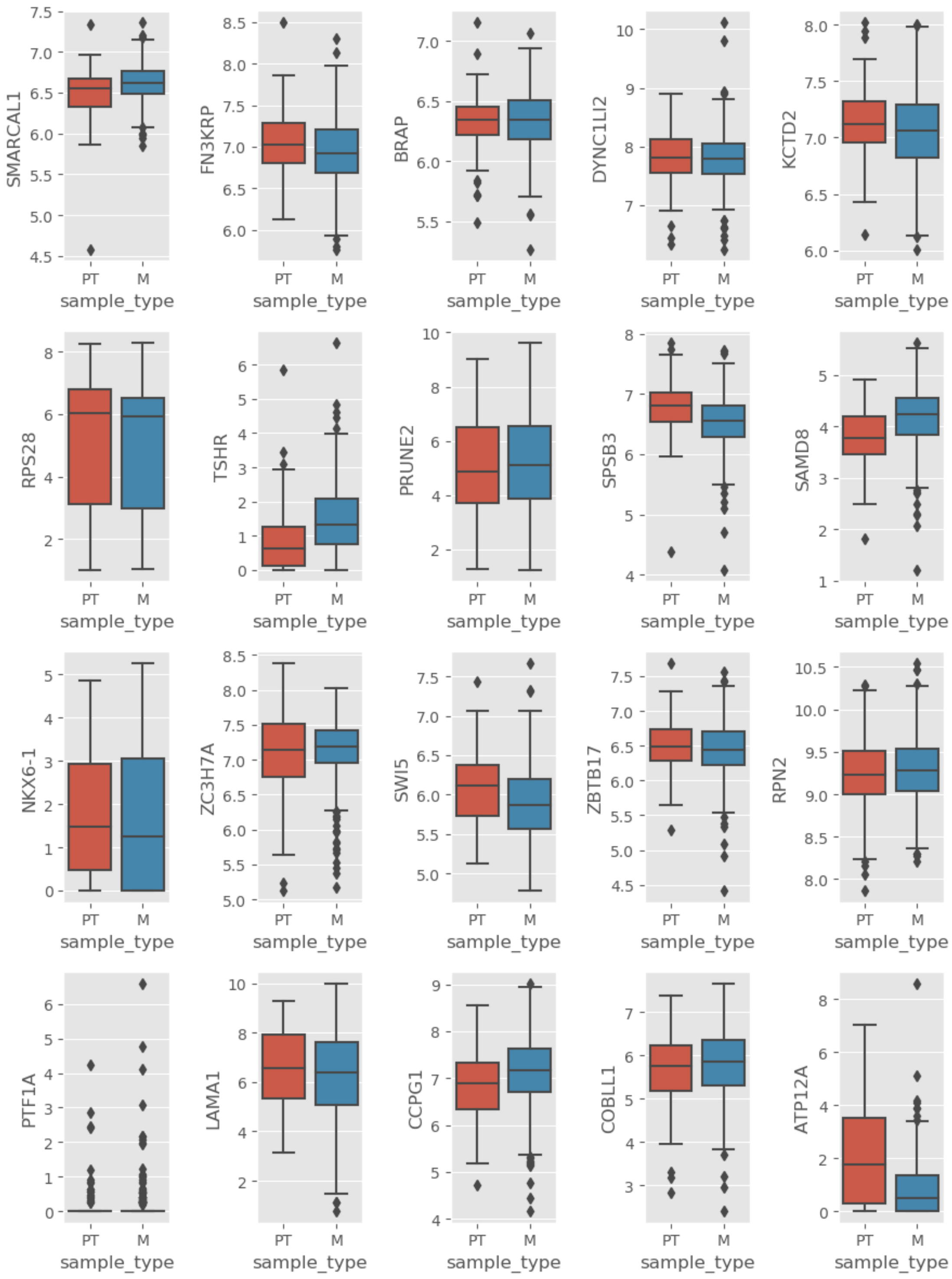

Figure S 39. Box Plots of Top 81 - 100 Gene Expressions.

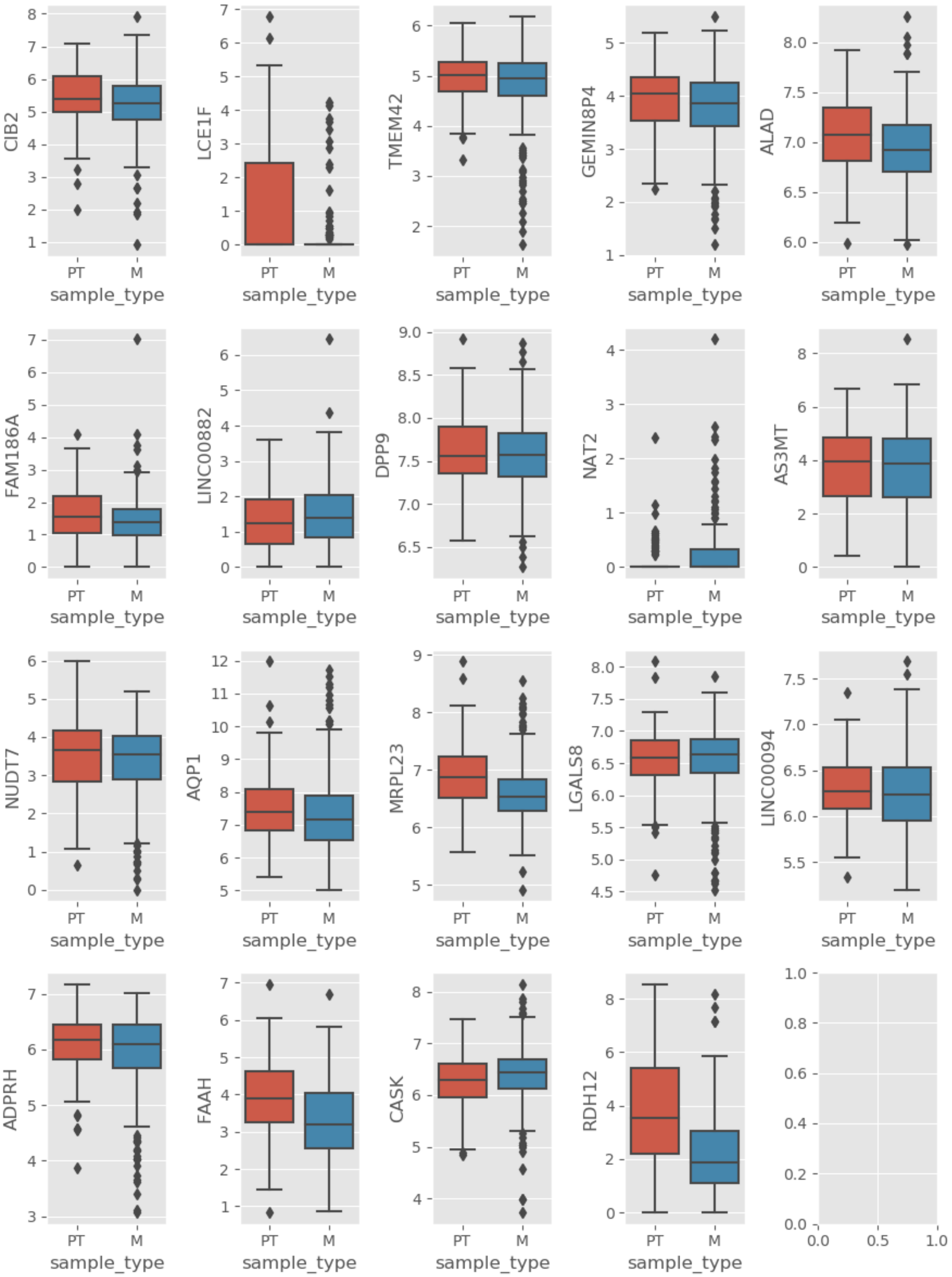

Figure S 40. Box Plots of Top 121 - 139 Gene Expressions.

Model	Average Expected Loss	Average Bias	Average Variance
RF-RF	0.0687	0.0569	0.0319
RF-LR	0.0933	0.0894	0.0311
RF-SVM-Sig	0.1202	0.1300	0.0275
RF-PPI-NB	0.1241	0.1219	0.0257
RF-NB	0.1378	0.1220	0.0704
RF-SVM-L	0.1407	0.1382	0.0069
RF-SVM-RB	0.1419	0.1382	0.0290
RF-PPI-LR	0.1423	0.1463	0.0149
RF-PPI-SVM-L	0.1463	0.1463	0.0295
RF-PPI-SVM-Sig	0.1951	0.2033	0.0100

Table S 1. Bias Variance Decomposition of the models

Model Name	F1	Accuracy	AUC
Ensemble Model with Soft Voting	90.60	91.05	0.9861

Table S 2. Model Performance (Validation Dataset) of Ensemble Model with Soft Voting
